# Laboratory animal ethics education improves medical students' awareness of laboratory animal ethics

**DOI:** 10.1186/s12909-024-05703-9

**Published:** 2024-07-01

**Authors:** Wang Zhang, Zhe Xie, Xue Fang, Zheng Wang, Zonghuan Li, Yulong Shi, Xinghuan Wang, Li Li, Xin Wang

**Affiliations:** 1 Department of Orthopedics Trauma and Microsurgery, Zhongnan Hospital of Wuhan University, Wuhan, Hubei 430071 China; 2 Division of Joint Surgery and Sports Medicine, Department of Orthopedic Surgery, Zhongnan Hospital of Wuhan University, Wuhan, 430071 Hubei China; 3 Department of Surgery, Second Clinical College, Wuhan University, Wuhan, 430071 Hubei China; 4 Department of Critical Care Medicine, Zhongnan Hospital of Wuhan University, Wuhan, 430071 Hubei China

**Keywords:** Medical students, Animal ethics, Education

## Abstract

**Objective:**

In this study, we added laboratory animal ethics education into both didactic sessions and practical sessions the general surgery laboratory course, with the didactic sessions focus on teaching the fundamental principles of laboratory animal ethics, while the practical sessions emphasize the application of these principles in laboratory classes and have assessed the changes in medical students' perception of laboratory animal ethics following medical students exposure to such education.

**Methods:**

One hundred and eighty-nine third-year medical students from Wuhan University's Second Clinical College completed a laboratory animal ethics awareness questionnaire and a laboratory animal ethics written examination before and after laboratory animal ethics education.

**Results:**

After receiving laboratory animal ethics education, the percentage of students who supported euthanasia for the execution of animals and humane treatment of laboratory animals were 95.2% and 98.8%, respectively, which did not differ from the 94.9% and 96.4% observed before the education. Moreover, there was a notable increase in the proportion of students who knew about regulations related to laboratory animals (from 39.9% to 57.1%), welfare issues (from 31.9% to 50.0%), and the 3R principle (from 30.4% to 58.9%) post-education, all statistically significant at *P* < 0.05. Test scores also showed improvement, with students scoring (93.02 ± 11.65) after education compared to (67.83 ± 8.08) before, a statistically significant difference.

**Conclusions:**

This research helps to provide information for the good practices of laboratory animal ethics education. After receiving laboratory animal ethics education, students are better able to treat laboratory animals in a correct animal ethical manner. Laboratory animal ethics education helps improve students' knowledge of laboratory animal ethics. Students’ perception towards how the laboratory animal ethics course should be delivered may vary. Still, new courses or better organized courses on laboratory animal ethics education are required in order to provide students an in-depth understanding.

**Supplementary Information:**

The online version contains supplementary material available at 10.1186/s12909-024-05703-9.

## Background

Laboratory animals are indispensable in many areas of the life sciences, contributing significantly to the advancement of scientific knowledge and the improvement of people's quality of life. Many people are concerned about the use of animals in medical research, and there has been a remerging emphasis on animal ethics problems in recent years [1–4]. In the 60 years since the publication of Principles of Humane Experimental Techniques, the 3R principles (Reduction, Refinement, Replacement, Reduction is a method of improving the efficiency of the use of experimental animals, using fewer animals to obtain the same amount of experimental data or using a certain number of animals to obtain more experimental data. Refinement refers to improving animal welfare by improving and refining procedures, avoiding inhumane methods, and alleviating or reducing the pain and distress caused to animals. Replacement refers to the search for alternatives, i.e. replacing animals with inanimate such as computer models and statistical analyses, or replacing higher vertebrates with less evolved vertebrates.) proposed by William Russell and Rex Birch have gradually gained acceptance around the world as a way to confront the ethical and scientific quandaries in animal experimentation [5]. This acceptance highlights the ongoing debate regarding the use of animals not only in medical research but also in medical education [6]. The social and moral rules and concepts that govern how laboratory animals and animal testing are referred to as laboratory animal ethics. Given this context, the concept of animal ethics no longer exists only for scientists but should also be introduced to a wider audience, such as university students [7]. This approach aligns with the recognition that ethics, including animal ethics, is an essential part of medical education [8, 9]. Implementing the 3R principles in laboratory animal ethics education will enhance medical students' ethical standards and practical skills in designing and conducting experiments. This will ultimately lead to better protection of animal welfare and enhanced quality of scientific research [10]. Currently, there is a substantial body of research on animal ethics, with a limited focus on laboratory animal ethics education specifically for medical students. The majority of existing studies in this area internationally concentrate on biomedical science and veterinary students [11–13], with fewer studies addressing laboratory animal ethics education for medical students in clinical medicine [14]. Moreover, there is a lack of systematic education in laboratory animal ethics. Educational gaps in laboratory animal ethics education have potential impact on medical practice. The insufficient emphasis on e laboratory animal ethics education among medical students could potentially result in a disregard for ethical standards in their future professional endeavors, impacting their decision-making and conduct in medical practice, this behavior has the potential to negatively impact both patients and the reputation of healthcare organizations [15, 16].A deficiency in ethics education may lead medical students to overlook animal welfare considerations during the design and implementation of experiments, consequently compromising the reliability and reproducibility of research outcomes, such compromised research findings could impede or misguide their application in medical practice [17, 18]. By enhancing medical students' awareness of animal welfare through ethics education, it is possible to alleviate the suffering and stress experienced by experimental animals, thereby obtaining more dependable experimental data essential for the advancement of drugs and treatments in medical practice [19, 20].Ultimately, the absence of ethics education may contribute to public skepticism towards scientific research and medical practice, influencing access to research funding and policy support, and potentially impeding medical advancements [21]. While medical ethics is a common course in medical schools in China, there is a lack of animal ethics education for medical students. In order to fill the gap in this area of laboratory animal ethics education, we added laboratory animal ethics education to the general surgery laboratory course. This study aims to evaluate the impact of this education on medical students' understanding of laboratory animal ethics.

## Methods

### Participants

One hundred and eighty-nine third-year medical students from Wuhan University's Second Clinical College were initially planned to be included to receive this laboratory animal ethics education. The selection and exclusion criteria for the samples were as follows: selection criteria: third-year medical students from the Second Clinical College of Wuhan University; exclusion criteria: those who did not receive laboratory animal ethics education; those who did receive education but did not complete the questionnaire and examination paper. There were one hundred and eighty-nine third -year medical students in our college, some of them did not participate in the questionnaire and question paper during the actual study, reasons for not being able to complete include, personal reasons: e.g. ill health, temporary family commitments and other non-research related personal reasons. Time conflict: e.g. conflict with other course schedules, resulting in the inability to complete the questionnaire and paper on time. Unwillingness to continue participation: some students find that they are not interested in the content or process of the study during their participation and choose to dropout, finally 73% (138/89) of the medical students completed the valid questionnaire and question paper before receiving the education on laboratory animal ethics. Following laboratory animal ethics education, 89% (168/189) of the medical students completed a valid questionnaire, while 73% (138/189) completed the examination paper. It is worth noting that these students had previously completed a general education and basic medical course, making this their first exposure to animal ethics education. This study was approved by the Research Ethics Committee of Zhongnan Hospital of Wuhan University(2022144K), and informed consent were obtained from all study participants, it was confirmed that they understood the purpose, process, possible risks and benefits of the study and participated voluntarily.

### Survey instrument

The items in the instrument are divided into two categories: a laboratory animal ethics awareness questionnaire and a laboratory animal ethics examination paper. The questionnaire and examination paper in this study were designed by considering current literature on laboratory animal ethics education and input from teachers, and were comprehensive in terms of medical students' attitudes towards laboratory animals, their knowledge of laboratory animal ethics, and their attitudes towards laboratory animal ethics education [22–24]. After the initial design, a panel of experts in medical education was consulted and pre-tested to ensure the reliability and validity of the survey instrument. The questionnaire has an introductory section: a brief explanation of the purpose of the survey, confidentiality and guidelines for completion. The main section comprised four modules: basic student information (e.g., gender, age), attitudes towards laboratory animals, knowledge on laboratory animal ethics, and attitudes towards laboratory animal ethics education. The survey was structured with an introductory section outlining the purpose, confidentiality, and completion guidelines. The main section comprised four modules: basic student information (e.g., gender, age), attitudes towards laboratory animals, knowledge on laboratory animal ethics, and attitudes towards laboratory animal ethics education. Multiple-choice options were provided throughout the questionnaire. The closing section included a thank you message and contact information. The exam papers are essentially questions that simulate experimental scenarios where students are asked to demonstrate how they would follow the code of animal ethics in their experiments. The laboratory animal ethics examination paper was set with a total of 10 questions, most of these were scenario based. The overall Cronbach's Alpha of the questionnaire obtained from the survey data was 0.730, indicating good reliability, and the KMO value of the questionnaire was 0.769, indicating good validity. The questionnaire and examination paper were distributed online through the Questionnaire Star platform (https://www.wjx.cn/). The questionnaires are all anonymous and offer no specific incentives.

### Study measures

**Fig. 1:**
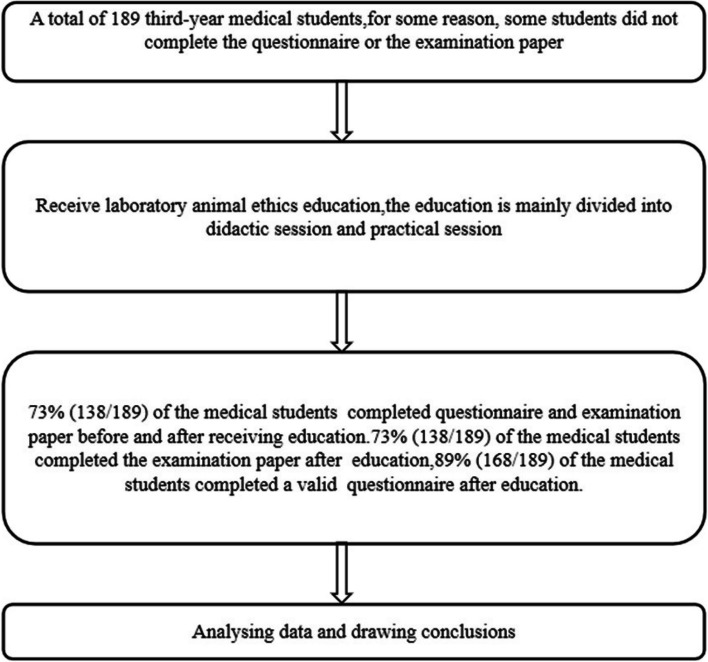
Illustrates the basic flow of the study design

Our intervention approach is to add laboratory animal ethics education to the general surgery laboratory course. Our learning objectives include cognitive, skill and attitude objectives. (Table 1 describes the above learning objectives) Learning objectives are assessed through questionnaire and examination paper, as well as feedback from teachers and students in practical sessions. The laboratory animal ethics education consists of two main components: a didactic session and a practical session. The didactic session, worth 1 credit hour, involves teachers delivering a PowerPoint presentation on animal ethics in the classroom. This part covers foundational concepts, the development process of laboratory animal ethics, the 3R principles, laws and regulations, welfare of laboratory animals, laboratory animal day, and the euthanasia treatment of animals. Following the didactic session, the practical session, worth 3 credit hours, takes place in an animal handling laboratory. Here, students independently conduct animal experiments while focusing on maintaining proper animal ethics. Emphasis is placed on handling animals gently, operating with care, avoiding cruelty, following standardized procedures for animal welfare, ensuring animals are adequately anesthetized during surgical procedures, and euthanizing animals at the end of the experiment in designated locations. The practical session emphasizes the importance of adhering to proper animal ethics during laboratory classes. The teacher will conclude by reviewing the content of animal experiments and addressing any instances of students' unethical behavior towards laboratory animals. Figure 1 illustrates the basic flow of the study design.

**Table 1:** Learning objectives of the intervention course

**Learning objectives**
**Cognitive objectives**
To recall the laws and regulations for the protection of laboratory animals and to describe the 3R principles.
**Skill objectives**
To correctly perform animal handling skills in grasping and fixing, correct anesthesia injection methods, culling of laboratory animals in order to reduce the pain of laboratory animals.
**Attitude objectives**
To treat laboratory animals in a correct animal ethical manner.

### Data analysis

When the statistical data were evaluated, those who answered “very necessary” and “necessary” in the questionnaire were classified as “support,” while those who answered “dispensable” and “not necessary” were classified as “not supported”. Furthermore, those who responded to the options “very aware” and “fairly aware” were classed as “aware” when the statistics were evaluated, while those who reacted to the options “heard of it but don’t know” and “don't know” were labeled as “I don’t know”. Count data were expressed as number of cases and percentage, Measurement data were expressed as (mean ± standard deviation), The chi-square test and Student’s t-test were utilized to compare categorical and continuous variables, respectively, between the groups. Non-parametric tests were employed when the sample did not adhere to a normal distribution or when significant outliers were present in the data. As a result, the Mann–Whitney U test was used to compare two independent samples that did not meet the criteria for a normal distribution. *P* < 0.05 was considered statistically significant. Data were analyzed using IBM SPSS 23.0 software.

## Results

### Results of the laboratory animal ethics awareness questionnaire

73% (138/189) of the medical students completed a valid survey before receiving laboratory animal ethics education, while 89% (168/189) completed a valid survey after the education. Prior to education, there were 138 students, 62 males and 76 females, with an average age of (20.78 ± 0.95) years. After education, there were a total of 168 students, 71 males and 97 females, with an average age of (20.71 ± 0.92) years. Statistical analysis showed no significant differences between the two groups in terms of gender (χ2 = 0.219, *P* = 0.640) and age (t = 0.638, *P* = 0.524).

### Attitudes towards laboratory animals before and after education

After receiving laboratory animal ethics education, the percentages of students who supported activities such as opposing increased suffering of laboratory animals due to improper handling and conducting silent mourning to laboratory animals were 94.1% and 91.7% respectively, an increase from 86.2% and 70.3% before education, and the difference was statistically significant (*P* < 0.05). The percentage of students who supported euthanasia of animals and humane treatment of laboratory animals after receiving laboratory animal ethics education was 95.2% and 98.8% respectively, this does not differ from the 94.9% and 96.4% observed before education. (Table 2).

**Table 2:** The laboratory animal ethics awareness questionnaire

Questions	Options	Pre	Post	*P* value*
		(*n* = 138)	(*n* = 168)	
**Attitudes towards laboratory animals before and after education**				
1. Whether it is necessary to oppose increased suffering of laboratory animals due to improper handling?	Support	119(86.2)	158(94.1)	< 0.05
	Not supported	19(13.8)	10(5.9)	
2. Whether to support euthanasia of animals?	Support	131(94.9)	160(95.2)	0.9
	Not supported	7(5.1)	8(4.8)	
3. Whether it is necessary to carry out activities such as silent mourning and memorials to laboratory animals?	Support	97(70.3)	154(91.7)	< 0.05
	Not supported	41(29.7)	14(8.3)	
4. Whether you support the humane treatment of laboratory animals?	Support	133(96.4)	166(98.8)	0.302
	Not supported	5(3.6)	2(1.2)	
**Awareness of knowledge related to ethics of laboratory animals before and after education**				
5. Whether you are aware of the relevant regulations for the protection of laboratory animals?	Understanding	55(39.9)	96(57.1)	< 0.05
	Don't know	83(60.1)	72(42.9)	
6. Whether you are aware of the welfare of laboratory animals?	Understanding	44(31.9)	84(50.0)	< 0.05
	Don't know	94(68.1)	84(50.0)	
7. Are you understanding of the 3R principles?	Understanding	42(30.4)	99(58.9)	< 0.05
	Don't know	96(69.6)	69(41.1)	
**Attitudes towards in laboratory animal ethics education before and after education**				
8. Whether you believe that strengthening animal ethics education is conducive to the development of good medical ethics?	Support	119(86.2)	167(99.4)	< 0.05
	Not supported	19(13.8)	1(0.6)	
9. Whether it is considered necessary for the school to offer courses related to ethics of laboratory animals?	Support	102(73.9)	133(79.2)	0.279
	Not supported	36(26.1)	35(20.8)	
10. Whether to support schools to use virtual simulation technology instead of animal experiments to reduce the number of animals used?	Support	84(60.9)	117(69.6)	0.108
	Not supported	54(39.1)	51(30.4)	

^*^Chi-square test; *Significant differences when *p*-value < 0.05

### Awareness of knowledge related to ethics of laboratory animals before and after education

A significantly higher proportion of students reported knowledge of regulations related to laboratory animals (57.1%) and welfare issues of laboratory animals (50.0%) after receiving laboratory animals ethics education than before receiving education (39.9%, *P* < 0.05, and 31.9%, *P* < 0.05, Table 3). The proportion of students who were aware of the 3R principles (58.9%) was also higher after receiving laboratory animal ethics education than before receiving education (30.4%, *P* < 0.05,Table 2)).

**Table 3:** Examination paper on laboratory animal ethics

Numbers	Questions
Q1	World Day for Laboratory Animals is celebrated on ()every year
Q2	If you were to euthanise a laboratory animal at the end of an animal experiment class, you would not choose ()
Q3	If you were taking a class on animal experimentation today, in order to comply with the requirements of basic surgical techniques for animal experimentation and observe the ethics of experimental animals, you would ()
Q4	Which of the following practices reflects the principle of "substitution" in the "3Rs" of animal ethics ()?
Q5	If you were an animal laboratory teacher and a class was to have an animal experiment lesson this morning, you would ()
Q6	If you see a student using a mobile phone to take photos of experimental animals in an animal experiment class and post them on the Internet, what will you do ()
Q7	Which of the following practices is not in line with the "five basic welfare" or "five freedoms" of animal ethics () ?
Q8	What would you do when you encounter a classmate in your neighbourhood who appears to be harming the animals purposelessly in the course of animal experiments ()
Q9	If you are conducting a laboratory class on appendectomy in rabbits, and you find that the rabbits show signs of resuscitation after anaesthesia, you would ()
Q10	If you are a small assistant in the animal laboratory of the university, in order to ensure the welfare of experimental animals, you will ()

### Attitudes towards in laboratory animal ethics education before and after education

After receiving laboratory animal ethics education, the proportion of students who thought that strengthening laboratory animal ethics education was conducive to cultivating good medical ethics was 99.4%, an increase from 86.2% before education, and the difference was statistically significant (*P* < 0.05). After receiving laboratory animal ethics education, the proportions of students who supported the introduction of courses related to laboratory animal ethics in schools and the adoption of virtual simulation experimental technology as an alternative to animal experiments in schools were 79.2% and 69.6%, respectively, this does not differ from the 73.9% and 60.9% observed prior to education. (Table 2).

### Results of the laboratory animal ethics examination paper

A total of 73% (138/189) of the medical students completed the examination paper before and after laboratory animal ethics education. The test scores of the medical students after the education were (93.04 ± 11.69) higher than that of the test scores before the education (67.83 ± 8.08), t = -20.85, *P* < 0.05, and the difference was statistically significant. (The content of the examination paper is in Table 3).

## Discussion

### Key Findings 

Given the importance of laboratory animal ethics in medical education, we created an innovative curriculum design for laboratory animal ethics education that consists of both didactic and practical modules that are integrated into the General Surgery laboratory course for third-year medical students. After receiving laboratory animal ethics education, students are better able to treat laboratory animals in a correct animal ethical manner. Laboratory animal ethics education helps improve students' knowledge of laboratory animal ethics. This is consistent with both the cognitive and attitudinal goals of animal ethics education. However, it is still not enough for students to have a full and comprehensive understanding, and more systematic courses about laboratory animal ethics education are needed. Students’ perception towards how the laboratory animal ethics course should be delivered may vary. Still, new courses or better organized courses on laboratory animal ethics education are required in order to provide students an in-depth understanding.

### Interpretation

After completing the education, there was a significant increase in the proportion of students who opposed improper handling and conducting silent mourning to laboratory animals. This change is likely because students were not aware that improper handling increased suffering for laboratory animals before they received education on animal ethics. And they were only made aware of the above events through a study of the current course and a questionnaire survey, which included the instructor in teaching about laboratory animal ethics during didactic session, as well as emphasizing the importance of treating laboratory animals kindly during practical session. This shows that there is still a need for our animal ethics education. Furthermore, a lack of understanding of laboratory animal ethics among medical students, who are future healthcare professionals, may tarnish their professional reputation and erode public trust. This is particularly concerning given the growing public interest in animal welfare within research [22, 25]. The percentage of students who knew about laboratory animal regulations, laboratory animal welfare issues, and the 3R principle increased significantly after receiving laboratory animal ethics education, indicating that current laboratory animal ethics education helped to improve students’ knowledge of laboratory animal ethics. The examination paper scores of medical students before and after the ethics education showed a significant difference, indicating the effectiveness of the education. Implementing animal ethics education at the undergraduate level has the potential to enrich students’ awareness of ethical implications and foster critical thinking skills in this area [26]. However, the percentage of students supporting the school's specialized laboratory animal ethics curriculum did not change significantly after the education. Before the education, 73.9% of students supported the introduction of laboratory animal ethics courses, which slightly increased to 79.2% afterward. This minimal change suggests that some medical students did not consider a formal curriculum was necessary for learning laboratory animal ethics and thought they could acquire this knowledge through other means. This suggests that future strategies for laboratory animal ethics education may be open to more options that do not lie solely in the curriculum, such as publishing knowledge related to laboratory animal ethics on the university's WeChat, official website and bulletin boards, and providing regular training and seminar opportunities for teachers and students to learn about the latest norms and standards of laboratory animal ethics [27].

### Comparison with Previous Studies

The results indicate that the proportion of students supporting euthanasia of animals and humane treatment of laboratory animals remained consistent before and after receiving laboratory animal ethics education. This suggests a high initial level of support that did not change, likely due to a ceiling effect. This phenomenon may benefit medical students in their future medical practice. The consistently high scores suggest that students already had a strong respect for life and a compassionate attitude towards living beings, which is in line with previous research [28, 29]. However, post-education data revealed that 42.9% of students were unaware of regulations related to laboratory animals, 50.0% were unfamiliar with animal welfare considerations, and 41.1% did not know about the 3R principles. These findings are consistent with earlier studies [30–32]. Medical knowledge and skills are undoubtedly the cornerstone of medical education; however, ethical principles also play a crucial role in the daily practice of doctors [33]. Many medical schools fail to provide students with the necessary guidance to develop ethical attitudes and apply their knowledge effectively [34]. The promotion of humanistic values and ethics in medicine is also considered a vital component of medical education [35–37]. Additionally, the findings showed that some students were motivated to learn about laboratory animal ethics at their schools and recognized the value of such courses, consistent with earlier research [25, 38, 39]. The information presented above implies that our findings are similar to prior research’ comparisons; for example, while most medical students are aware of animal ethics, they do not have sufficient knowledge of experimental animal ethics. In addition, and this is new to us, we have found that students’ perception towards how the laboratory animal ethics course should be delivered may vary. However, it is worth noting that, the proportion of students supporting the use of virtual simulation as an alternative to animal experimentation remained unchanged after the education, this is consistent with previous findings [40]. This is most likely due to the fact that students find traditional teaching methods easier to remember new knowledge and technical skills. Virtual simulation-based learning aligns with the “substitution” principle in animal use ethics, and knowledge of the 3Rs and alternatives should be part of the education and training process [41, 42]. Therefore, a blend of traditional and alternative teaching approaches could be employed in future education to enhance learning outcomes.

### Implications

This indicates that while the students have some background in medical ethics, their knowledge of laboratory animal ethics still needs improvement. Given the complexity of this field, it may be challenging for students to fully grasp in a short timeframe. This suggests that medical students might benefit from additional, dedicated sessions on laboratory animal ethics in the future. Laboratory animal ethics education has an impact on practice because it not only raises medical students' ethical consciousness and improves the welfare of laboratory animals, but it also has a beneficial and far-reaching impact on the quality of current research. Implications for future research include promoting ethical research development, disseminating the 3R principles, and encouraging international collaboration. Medical schools should consider offering mandatory courses on laboratory animal ethics or integrating relevant animal ethics laws and guidance documents into existing medical ethics and professional courses related to animal experimentation.

## Limitations

Our study provides an innovative approach to delivering laboratory animal ethics education to medical students. Despite these strengths, the study does have some limitations: 1) As a single-center study, the findings may not be easily generalized. Future interventions should involve multiple centers to enhance the assessment of effectiveness and applicability. 2) The study lacked a comparison between students who received animal ethics education and a control group of students from the same cluster who did not receive such training. Furthermore, there were no comparisons with previous cohorts to evaluate changes in opinions and knowledge. A randomized controlled trial may be necessary for future research to address these limitations. 3) The questionnaire design may not have fully explored the range of students’ perspectives and understanding of animal ethics issues. Future studies could incorporate more detailed and diverse methodologies, such as in-depth interviews or group discussions, to gain deeper insights. 4) The study had a limited scope and did not track changes in students' knowledge and attitudes over time. To fully grasp the enduring impact of educational interventions, future research should include long-term follow-up.

## Conclusions

This study investigates the impact of incorporating laboratory animal ethics education into a general surgery laboratory course for medical students, setting the stage for future advancements in this field. The results suggest that such education improves students' ability to ethically care for laboratory animals and enhances their comprehension and awareness of animal ethics. However, there is still room for improvement in their overall understanding of laboratory animal ethics. Furthermore, students have diverse opinions on the structure of the ethics program. Therefore, refining the educational content and promoting laboratory animal ethics education is crucial for enhancing medical students’ ethical awareness and knowledge in this area. It is recommended that curriculum developers in other institutions consider offering courses on laboratory animal ethics and making them mandatory. Alternatively, integrating the content of relevant animal ethics laws from different countries and animal ethics guidelines from international organizations into existing medical ethics and professional courses involving animal experiments could be beneficial.

### Supplementary Information

Supplementary Material 1.

## Data Availability

All data are incorporated into the article and its online supplementary material. The data underlying this article are available in the article and in its online supplementary material.
