# Supplementary material for: Laboratory animal ethics education improves medical students' awareness of laboratory animal ethics
Source: BMC Med Educ. 2024 Jul 1;24:709. doi: 10.1186/s12909-024-05703-9 (PMC11218205; doi:10.1186/s12909-024-05703-9)
Supplement: Supplementary file 1 — Supplementary Material 1. [file 12909_2024_5703_MOESM1_ESM.zip › Questionnaire survey data.pdf]

Question 1

个案处理摘要

	个案					
	有效		缺失		总计	
	N	百分比	N	百分比	N	百分比
组别 * 态度	306	100.0%	0	0.0%	306	100.0%

组别 * 态度 交叉表

计数

		态度		总计
		支持	不支持	
组别	2020级前	119	19	138
	2020级后	158	10	168
总计		277	29	306

卡方检验

	值	自由度	渐进显著性（双侧）	精确显著性（双侧）	精确显著性（单侧）
皮尔逊卡方	5.395[a]	1	.020		
连续性修正[b]	4.522	1	.033		
似然比	5.403	1	.020		
费希尔精确检验				.030	.017
线性关联	5.377	1	.020		
有效个案数	306				

a. 0 个单元格 (.0%) 的期望计数小于 5。最小期望计数为 13.08。

b. 仅针对 2x2 表进行计算

Question 2

个案处理摘要

	个案					
	有效		缺失		总计	
	N	百分比	N	百分比	N	百分比
组别 * 态度	306	100.0%	0	0.0%	306	100.0%

组别 * 态度 交叉表

计数

		态度		总计
		支持	不支持	
组别	2020级前	131	7	138
	2020级后	160	8	168
总计		291	15	306

卡方检验

	值	自由度	渐进显著性（双侧）	精确显著性（双侧）	精确显著性（单侧）
皮尔逊卡方	.016[a]	1	.900		
连续性修正[b]	.000	1	1.000		
似然比	.016	1	.900		
费希尔精确检验				1.000	.553
线性关联	.016	1	.901		
有效个案数	306				

a. 0 个单元格 (.0%) 的期望计数小于 5。最小期望计数为 6.76。

b. 仅针对 2x2 表进行计算

Question 3

[数据集0]

个案处理摘要

	个案					
	有效		缺失		总计	
	N	百分比	N	百分比	N	百分比
组别 * 态度	306	100.0%	0	0.0%	306	100.0%

组别 * 态度 交叉表

计数

		态度		总计
		支持	不支持	
组别	2020级前	97	41	138
	2020级后	154	14	168
总计		251	55	306

卡方检验

	值	自由度	渐进显著性（双侧）	精确显著性（双侧）	精确显著性（单侧）
皮尔逊卡方	23.483[a]	1	.000		
连续性修正[b]	22.056	1	.000		
似然比	23.958	1	.000		
费希尔精确检验				.000	.000
线性关联	23.407	1	.000		
有效个案数	306				

a. 0 个单元格 (.0%) 的期望计数小于 5。最小期望计数为 24.80。

b. 仅针对 2x2 表进行计算

Question 4

个案处理摘要

	个案					
	有效		缺失		总计	
	N	百分比	N	百分比	N	百分比
组别 * 态度	306	100.0%	0	0.0%	306	100.0%

组别 * 态度 交叉表

计数

		态度		总计
		支持	不支持	
组别	2020级前	133	5	138
	2020级后	166	2	168
总计		299	7	306

卡方检验

	值	自由度	渐进显著性（双侧）	精确显著性（双侧）	精确显著性（单侧）
皮尔逊卡方	2.006[a]	1	.157		
连续性修正[b]	1.065	1	.302		
似然比	2.032	1	.154		
费希尔精确检验				.250	.151
线性关联	1.999	1	.157		
有效个案数	306				

a. 2 个单元格 (50.0%) 的期望计数小于 5。最小期望计数为 3.16。

b. 仅针对 2x2 表进行计算

Question 5

个案处理摘要

	个案					
	有效		缺失		总计	
	N	百分比	N	百分比	N	百分比
组别 * 态度	306	100.0%	0	0.0%	306	100.0%

组别 * 态度 交叉表

计数

		态度		总计
		支持	不支持	
组别	2020级前	55	83	138
	2020级后	96	72	168
总计		151	155	306

卡方检验

	值	自由度	渐进显著性（双侧）	精确显著性（双侧）	精确显著性（单侧）
皮尔逊卡方	9.059[a]	1	.003		
连续性修正[b]	8.381	1	.004		
似然比	9.109	1	.003		
费希尔精确检验				.003	.002
线性关联	9.029	1	.003		
有效个案数	306				

a. 0 个单元格 (.0%) 的期望计数小于 5。最小期望计数为 68.10。

b. 仅针对 2x2 表进行计算

Question 6

个案处理摘要

	个案					
	有效		缺失		总计	
	N	百分比	N	百分比	N	百分比
组别 * 态度	306	100.0%	0	0.0%	306	100.0%

组别 * 态度 交叉表

计数

		态度		总计
		支持	不支持	
组别	2020级前	44	94	138
	2020级后	84	84	168
总计		128	178	306

卡方检验

	值	自由度	渐进显著性（双侧）	精确显著性（双侧）	精确显著性（单侧）
皮尔逊卡方	10.219[a]	1	.001		
连续性修正[b]	9.488	1	.002		
似然比	10.328	1	.001		
费希尔精确检验				.002	.001
线性关联	10.185	1	.001		
有效个案数	306				

a. 0 个单元格 (.0%) 的期望计数小于 5。最小期望计数为 57.73。

b. 仅针对 2x2 表进行计算

Question 7

个案处理摘要

	个案					
	有效		缺失		总计	
	N	百分比	N	百分比	N	百分比
组别 * 态度	306	100.0%	0	0.0%	306	100.0%

组别 * 态度 交叉表

计数

		态度		总计
		支持	不支持	
组别	2020级前	42	96	138
	2020级后	99	69	168
总计		141	165	306

卡方检验

	值	自由度	渐进显著性（双侧）	精确显著性（双侧）	精确显著性（单侧）
皮尔逊卡方	24.758[a]	1	.000		
连续性修正[b]	23.624	1	.000		
似然比	25.207	1	.000		
费希尔精确检验				.000	.000
线性关联	24.677	1	.000		
有效个案数	306				

a. 0 个单元格 (.0%) 的期望计数小于 5。最小期望计数为 63.59。

b. 仅针对 2x2 表进行计算

Question 8

个案处理摘要

	个案					
	有效		缺失		总计	
	N	百分比	N	百分比	N	百分比
组别 * 态度	306	100.0%	0	0.0%	306	100.0%

组别 * 态度 交叉表

计数

		态度		总计
		支持	不支持	
组别	2020级前	119	19	138
	2020级后	167	1	168
总计		286	20	306

卡方检验

	值	自由度	渐进显著性（双侧）	精确显著性（双侧）	精确显著性（单侧）
皮尔逊卡方	21.522[a]	1	.000		
连续性修正[b]	19.419	1	.000		
似然比	24.934	1	.000		
费希尔精确检验				.000	.000
线性关联	21.451	1	.000		
有效个案数	306				

a. 0 个单元格 (.0%) 的期望计数小于 5。最小期望计数为 9.02。

b. 仅针对 2x2 表进行计算

Question 9

个案处理摘要

	个案					
	有效		缺失		总计	
	N	百分比	N	百分比	N	百分比
组别 * 态度	306	100.0%	0	0.0%	306	100.0%

组别 * 态度 交叉表

计数

		态度		总计
		支持	不支持	
组别	2020级前	102	36	138
	2020级后	133	35	168
总计		235	71	306

卡方检验

	值	自由度	渐进显著性（双侧）	精确显著性（双侧）	精确显著性（单侧）
皮尔逊卡方	1.174[a]	1	.279		
连续性修正[b]	.897	1	.344		
似然比	1.169	1	.280		
费希尔精确检验				.341	.172
线性关联	1.170	1	.279		
有效个案数	306				

a. 0 个单元格 (.0%) 的期望计数小于 5。最小期望计数为 32.02。

b. 仅针对 2x2 表进行计算

Question 10

个案处理摘要

	个案					
	有效		缺失		总计	
	N	百分比	N	百分比	N	百分比
组别 * 态度	306	100.0%	0	0.0%	306	100.0%

组别 * 态度 交叉表

计数

		态度		总计
		支持	不支持	
组别	2020级前	84	54	138
	2020级后	117	51	168
总计		201	105	306

卡方检验

	值	自由度	渐进显著性（双侧）	精确显著性（双侧）	精确显著性（单侧）
皮尔逊卡方	2.587[a]	1	.108		
连续性修正[b]	2.213	1	.137		
似然比	2.582	1	.108		
费希尔精确检验				.117	.069
线性关联	2.579	1	.108		
有效个案数	306				

a. 0 个单元格 (.0%) 的期望计数小于 5。最小期望计数为 47.35。

b. 仅针对 2x2 表进行计算
