# Supplementary material for: Laboratory animal ethics education improves medical students' awareness of laboratory animal ethics
Source: BMC Med Educ. 2024 Jul 1;24:709. doi: 10.1186/s12909-024-05703-9 (PMC11218205; doi:10.1186/s12909-024-05703-9)
Supplement: Supplementary file 1 — Supplementary Material 1. [file 12909_2024_5703_MOESM1_ESM.zip › The revised table/Knowledge and Opinions of Third Year Veterinary Students Relevant to Animal Welfare Before and After Implementation of a Core Welfare Course.pdf]

frontiers in Veterinary Science

ORIGINAL RESEARCH
published: 10 April 2019
doi: 10.3389/fvets.2019.00103

Knowledge and Opinions of Third Year Veterinary Students Relevant to Animal Welfare Before and After Implementation of a Core Welfare Course

Elizabeth C. S. Johnstone[1], *Melinda A. Frye*[2], *Linda K. Lord*[3], *Angela K. Baysinger*[4] and *Lily N. Edwards-Callaway*[1]*

[1] Department of Animal Sciences, Colorado State University, Fort Collins, CO, United States, [2] College of Veterinary Medicine and Biomedical Sciences, Colorado State University, Fort Collins, CO, United States, [3] Veterinary and Consumer Affairs, Merck Animal Health, Madison, NJ, United States, [4] Veterinary and Consumer Affairs, Merck Animal Health, De Soto, KS, United States

OPEN ACCESS

Edited by:
Emily Patterson-Kane,
American Veterinary Medical Association, United States

Reviewed by:
Orla Shortall,
James Hutton Institute, United Kingdom
Vera Baumans,
Utrecht University, Netherlands

***Correspondence:**
Lily N. Edwards-Callaway
lily.edwards-callaway@colostate.edu

Specialty section:
This article was submitted to Veterinary Humanities and Social Sciences, a section of the journal Frontiers in Veterinary Science

Received: 22 January 2019
Accepted: 19 March 2019
Published: 10 April 2019

Citation:
Johnstone ECS, Frye MA, Lord LK, Baysinger AK and Edwards-Callaway LN (2019) Knowledge and Opinions of Third Year Veterinary Students Relevant to Animal Welfare Before and After Implementation of a Core Welfare Course. Front. Vet. Sci. 6:103. doi: 10.3389/fvets.2019.00103

Although leading veterinary organizations emphasize the importance of animal welfare knowledge, there exists a gap in current veterinary student animal welfare education and training. A survey instrument was created to assess third-year Doctor of Veterinary Medicine (DVM) student knowledge of key animal welfare topics, opinions regarding the inclusion of welfare education in the veterinary curriculum, and views on veterinarian responsibilities as advocates. In Spring 2018, Colorado State University added a required animal welfare course to the DVM curriculum. Pre- and post-course paper surveys were distributed to the third-year students enrolled in the animal welfare course. One hundred thirty one completed pre-course surveys were collected and 125 completed post-course surveys were collected. Of the pre and post-course surveys collected, 61 were paired with identification codes and utilized for statistical comparison. Results indicated that the course led students to view the inclusion of an animal welfare course in the veterinary curriculum more favorably ($p = 0.009$) and improved their confidence in conducting research on animal welfare topics ($p < 0.001$). The course did not change students' sense of responsibility toward welfare advocacy. Associations were not found between attitudes toward these issues and demographic variables of home community, respondent gender, and track selection ($p > 0.06$). Veterinarians were consistently ranked by students as the most influential member of a community in matters of animal welfare. Future research on the lack of veterinary student knowledge of animal welfare should be done on a national scale to facilitate strategic development of mandatory animal welfare courses in veterinary curricula. Future research should be designed to gain knowledge regarding DVM students' opinions and attitudes regarding effective methods of incorporating animal welfare education into their professional training.

Keywords: advocate, animal welfare, attitudes, curriculum, veterinary student

INTRODUCTION

Protecting the welfare of animals, whether they are animals produced for food, companions, or research subjects, is a human responsibility. While the obligation to care for animals is universally shared, veterinarians are often considered the primary advocates for animals and the ultimate champions of their well-being (1–3). Upon graduating from training, veterinarians globally pledge not only to uphold, but to promote the principles of animal welfare for the benefit of animal well-being and society (4).

In the past few decades, public demand for greater consideration of animal well-being across animal industries has strengthened the need for veterinarians to have current and broad knowledge of issues and trends in animal welfare, regardless of veterinary specialty (5, 6). At the same time, leading organizations in animal health and welfare, such as the American Veterinary Medical Association (AVMA) and the American Association of Veterinary Medical Colleges (AAVMC), emphasize the necessity for veterinarians to be sufficiently knowledgeable about animal welfare science and issues in order to be effective change-makers; indeed, veterinarians must continually seek to enhance, improve and evolve their knowledge and opinions regarding animal health and welfare (7). To fulfill this obligation to protect animal welfare, as sworn in the Veterinarian's Oath upon graduation, proper training must be offered by veterinary programs in the scientific study of animal welfare (8).

The AVMA identifies a "noticeable gap" between the goal of Doctor of Veterinary Medicine (DVM) education in preparing students to be proactive and effective advocates for animal welfare and the actuality of that occurring given the lack of animal welfare courses in veterinary schools nationwide (9, 10). The AVMA Council on Education (COE) requires veterinary curriculums of veterinary colleges eligible for AVMA accreditation to provide "knowledge, skills, values, attitudes, aptitudes, and behaviors necessary to address responsibly the health and well-being of animals in the context of ever-changing societal expectations" but any mention of training in animal welfare science is absent from these listed requirements (11).

The United States (US) lags behind Europe and Latin America in implementing welfare education in veterinary schools (2). A 2016 study, in which a curricular review was performed across the 30 AVMA-accredited mainland US veterinary schools, found that only six offered a formal, 1 to 2 credit course that included the term animal welfare in the title with an inconsistent variety of species and topics covered (10). As of 2018, one author of this paper (ECSJ), in searching veterinary curriculums for courses with either "welfare" in the title or course descriptions containing central themes of welfare education, found that only nine of the 30 mainland AVMA-accredited US veterinary schools currently offer a formal course on animal welfare. Most courses indicated in the course descriptions that the instruction focused on general animal welfare without a particular emphasis on species. A required animal welfare course would, at the very least, establish a baseline from which veterinary knowledge and management of animal welfare issues can be measured as DVM students move from training to professional practice (3, 10, 12).

In the last 20 years, several surveys have been conducted to better understand veterinary students' capacity for empathy toward animal suffering, pain, and overall compromised well-being; their knowledge of welfare issues; and their attitudes toward animal welfare education (13–18). Surveys analyzing responses by DVM students enrolled in animal welfare courses have generally found that the courses have effectively challenged students to improve their ability to identify compromised welfare, discuss solutions and encouraged ethical considerations (15, 17). A 2010 study of DVM students revealed that an elective animal welfare course promoted favorable opinions toward the prospective inclusion of such a course in the required curriculum, improved knowledge of welfare evaluation criteria and strategies, and promoted confidence in self-educating about animal welfare topics (15).

Only one of these studies, conducted in the US, focused on a required, vs. elective, animal welfare course (17). The survey assessed student perception of such a course and was administered to first year DVM students following completion of the mandatory two-credit course. While the survey highlighted the positive reception of an animal welfare course by first year students, it did not evaluate possible changes in student knowledge of, and opinions toward, animal welfare and animal welfare advocacy as a result of completing the required course.

The purpose of this study was to examine the current opinions of third-year veterinary students, never before exposed to a professional degree animal welfare course, toward the implementation of such a required course; to assess these veterinary students' opinions toward their roles within a community in making animal welfare decisions; to assess the confidence of these veterinary students in educating themselves about animal welfare issues; and to assess the effect of this new course on changing students' knowledge of and opinions toward their roles as animal welfare advocates.

MATERIALS AND METHODS

The College of Veterinary Medicine and Biomedical Sciences (CVMBS) at CSU introduced an animal welfare course in the third-year veterinary curriculum in the spring of 2018. The course was approved by the University Curriculum Committee in the Fall of 2017 as a required element of the third-year, second semester DVM curriculum. The animal welfare course, which met twice weekly for a total of 2 h, was designed to introduce students to the basic principles of animal welfare science through lectures, discussions, professional panels, and student assignments. The course was developed using guidance from the model curriculum put forth by the AVMA Model Animal Welfare Curriculum Planning Group (19).

General

Survey questions regarding third-year DVM students' attitudes toward, and knowledge of, animal welfare topics, animal welfare

education, and their responsibilities as animal welfare advocates were developed by individuals within the CSU Department of Animal Science and the CVMBS. The survey was tested by two graduate students within CSU's Animal Science department and two veterinarian mentors to one of the co-authors of this paper (ECSJ). This survey was examined by the Institutional Review Board at CSU and deemed exempt from full board review. A paper survey was developed and administered in-person to veterinary students ($N = 145$) enrolled in the two-credit animal welfare course. A pre-course survey and an identical (except for demographic information) post-course survey was administered to the course registrants. On January 19, 2018, the paper pre-course survey was administered to students of the first day of the animal welfare course by one of the co-authors (ECSJ). Students were verbally informed of the voluntary nature of this survey and no incentives were provided. Students were informed that by returning the blank survey they could opt out of participation and their consent to participate would be given by returning a completed survey. Each survey had an informed consent cover page attached in front of the survey questions that repeated these details and provided further information of informed consent and the appropriate individuals to contact with any concerns or questions. After being informed of the anonymous nature of the survey, respondents were asked to provide a "Survey ID code" which consisted of the last two letters of their mother's maiden name and the first three numbers of their hometown zip code. This identifier was used to match pre- and post-course surveys and assess change in individual responses. Surveys were collected in person by one of the authors of this paper (ECSJ) following completion. A total of 131 students completed the survey.

On April 25, 2018, the same survey was administered to the students on the final day of class using an identical method of administration and informed consent as that of the first survey. As planned, the post-course survey was modified by removing the demographic questions. One additional question was removed from the post-course survey as pre-course responses indicated that students did not consistently answer the question in the manner intended (i.e., rating vs. ranking). Respondents were asked to include the same Survey ID code on the post-course survey as was provided on the pre-course survey. The post-course survey was collected from voluntary respondents ($n = 125$) by one of the authors of this paper (ECSJ) at the end of the last class session after completion.

The first section in the pre-course survey consisted of demographic questions of age, gender, race or ethnicity, home community, and dietary preference (**Table 1**). For each demographic question apart from age, respondents were given the choice of selecting "Not Defined" and a write-in option was provided if they did not identify with the categories listed. **Table 1** also includes specialization and curriculum track responses (Q1 and Q2 on the survey). Pre-course surveys that did not have ID codes provided were given a unique identifier and were included only in the pre-course data analysis for summary statistics ($n = 15$).

TABLE 1 | Summary of survey respondent demographics collected from the pre-course survey (% of total respondents (*n*); $N = 131^a$).

Demographic	% of respondents (*n*)[a]
**AGE**	
20–24	26.0 (34)
25–29	54.2 (71)
30–34	16.0 (21)
35–39	1.5 (2)
40–44	1.5 (2)
45–49	0.0 (0)
50+	0.8 (1)
**HOME COMMUNITY**	
Suburban	57.3 (75)
Rural	22.9 (30)
Urban	18.3 (24)
Not Defined	1.5 (2)
**TRACK**	
Small animal	54.2 (71)
General	26.7 (35)
Large animal	19.1 (25)
**GENDER**	
Female	84.0 (110)
Male	16.0 (21)
**PRACTICE INTEREST**	
Companion animal	52.7 (69)
Mixed	26.0 (34)
Research/academia	20.6 (27)
Exotics/zoo med	13.0 (17)
Equine	11.5 (15)
Food animal	9.2 (12)
Public health/policy	6.9 (9)
Wildlife	6.1 (8)
Lab animal	2.3 (3)
**DIETARY PREFERENCE**	
Non-vegetarian	88.5 (116)
Vegetarian	7.6 (10)
Vegan	1.5 (2)
Not defined	2.2 (3)

[a]*Total pre-course responses ($n = 131$), ID code pairing was not considered in demographic summary.*

The remainder of the survey consisted of 22 questions (pre-course) and 21 questions (post-course) consisting of both Likert scale, ranking and binary response questions. For the purposes of this paper, four questions were selected for analysis (**Table 2**). The focus of this paper is on attitudes toward animal welfare education and advocacy and therefore the questions related to those specific topics were chosen for inclusion. The additional questions from the survey not included here focused on specific welfare issues (e.g., pain mitigation), metrics used to assess welfare and their significance, and concern for the welfare of different categories of animals (e.g., food animal, exotics, companion, etc.). Data from additional questions will be reviewed in subsequent papers.

Quantitative Analysis

Pre-course and post-course survey data was manually entered into Numbers[1] software. Only completed surveys with all questions answered were included in both the pre- and post-course analysis. Demographic data was summarized by calculating percentages for all demographic categories using Numbers[1] software. Data from the pre-course survey ($n = 131$) questions and the post-course survey ($n = 125$) questions with Likert scale and ranking responses were summarized. Mean Likert scale responses and mean ranking responses were summarized using R software.[2] Percentages of responses greater than a Likert value of 3 (indicating agreement) were summarized using Numbers[1] software. In this way, Likert scale responses were made binary by treating responses >3 as "Yes/Agree" and responses 3 or less as "No/Disagree." For the pre-course survey data analysis, Fisher Exact Tests were used to analyze associations between demographic variables and Likert scale questions (converted to binary) using R software.[2]

More than half of the survey ID codes written by respondents on the post-course surveys did not match the ID codes given by respondents on the pre-course surveys. The authors of this paper do not know what caused the discrepancies between ID codes and these errors are unfortunate in that they limited the statistical analysis that could be performed. Due to these errors, only 61 completed surveys could be paired by ID codes and used for comparative statistical testing. The total group of post-course survey respondents ($n = 125$) cannot be said to be the same group as the pre-course survey respondents ($n = 131$) and may contain a proportion of students that did not complete pre-course surveys, thereby making the total post-course sample a different combination of individuals from the pre-course sample. Therefore, statistical testing was not used to compare total pre-course and total post-course response data but summary statistics on these two populations are included for consideration.

For the paired ($n = 61$) survey questions with Likert scale and ranking responses, the significant differences between the mean Likert scale responses or the mean ranking response for individual questions were tested by paired t-tests using R software[2]. For the same paired survey questions with Likert responses, significant differences between the proportion of responses with Likert values >3 (Likert scale converted to a binary response of agree/disagree) in the pre and post-course data were tested by McNemar's test using R software[2].

Statistical significance was designated a priori for all tests performed as $p \leq 0.05$.

Qualitative Analysis

Qualitative thematic analysis was performed on Question 3 (Q3) of both the pre-course and post-course survey. Question 3 asked respondents to respond to the statement "It is important to have an animal welfare and ethics course as part of the veterinary curriculum" using a Likert scale with a follow-up write-in response asking "Why or why not?" (**Table 2**). These write-in responses were transcribed into Numbers[1] software for both the pre-course surveys and post-course surveys. Write-in responses paired by ID codes ($n = 30$) were used to compare proportional changes in attitudes from pre-course and post-course. Summary statistics were also performed on the total write-in responses for the pre and post-course surveys ($n = 93$ and $n = 79$, respectively), with the acknowledgment that these responses would be treated as samples from distinctly separate populations of students. Thematic codes were created by a co-author based on preliminary review of all responses. Many different opinions were expressed in these written responses but there were common themes that became apparent when sorting through all the responses. The co-author created thematic categories based on these common themes as a means of organizing the written responses into groups that contained similar opinions and ideas that could then be statistically compared. Two graduate students, one being a co-author (ECSJ), coded the write-in responses to Q3 for both the completed pre-course surveys and post-course surveys. Biases were established verbally before coding to eliminate the tendency of either coder to code responses based on personal bias resulting from previous experience or knowledge. Both coders verbalized to one another any personal bias they were aware of that related to the research subject and the study participants in an in-person discussion before coding began. The average percent agreement between the two coders for all survey responses was 92.5%. This value was calculated by comparing the two codes assigned to each response (for the paired responses and the total pre and total post responses) one from each coder, and dividing the total responses ($n = 30$, $n = 93$, $n = 79$) by the number of responses to which identical codes were assigned. The three percentages generated by these calculations were then averaged to give a final percent agreement value.

RESULTS

The pre-course survey response rate was 90.3% ($n = 131$). The post-course survey response rate was 86.2% ($n = 125$). Only 61 pre- and post-course surveys were able to be paired (41.2% response rate) and used for statistical testing. Summary statistics for the total pre- and post-course survey responses are provided in a selection of tables and generally indicate similar results to the paired responses.

Demographics

Of the 131 individuals who completed pre-course surveys, the majority of respondents ($n = 105$; 80.2%) were between the ages of 20 and 29, 17.5% ($n = 23$) of individuals were between 30 and 39 years old, and 2.3% ($n = 3$) of individuals were over the age of 40 (**Table 1**). Eighty four percent ($n = 110$) of respondents were female and 16.0% ($n = 21$) of respondents were male. More than half ($n = 75$; 57.3%) of respondents identified growing up in suburban communities while 22.9% ($n = 30$) of respondents identified growing up in rural communities and 18.3% ($n = 24$) identified growing up in urban communities. Regarding program track, 54.2% ($n = 71$) of respondents had selected to track small animal medicine, 26.7% ($n = 35$) had selected to track general medicine, and 19.1% ($n = 25$) had selected to track large animal medicine. When asked about their

[1] Apple Numbers, 2018, Apple Inc., Cupertino, CA.
[2] R Software, version 1.1.383, R Core Team, Vienna, Austria.

TABLE 2 | Selected survey questions used for analysis focusing on veterinary student attitudes toward animal welfare education and advocacy.

Survey question ID	Question	Response type
Q3	It is important to have an animal welfare and ethics course as part of the veterinary curriculum	Likert scale (1 = strongly disagree; 5 = strongly agree)<br>Place for written comments
Q6	I feel confident that I know how to research an animal welfare topic, even one that I know very little about, in order to form an educated opinion that I can communicate to others	Likert scale (1 = strongly disagree; 5 = strongly agree)
Q11	How influential should the listed individuals be in making animal welfare decisions within a community?<br>Animal rights organizations/campaigners<br>Politicians<br>Animal scientists<br>General public<br>Animal industry members<br>Veterinarians	Ranking for each option (1 = highly influential; 5 = minimally or not at all influential)
Q13	As an expert in a particular animal type, I am obligated to be an advocate for the welfare of all animals in my community	Likert scale (1 = strongly disagree; 5 = strongly agree)

TABLE 3 | Total pre-course[a], total post-course[b], and paired[c] (by matched ID codes) survey responses to questions containing Likert Scale responses.

Survey question	Overall mean Likert* response (± SEM)					Proportion of respondents with Likert values >3 (*n*)				
	Pre-course (*N* = 131)	Post-course (*N* = 125)	Paired (*N* = 61)			Pre-course (*N* = 131)	Post-course (*N* = 125)	Paired (*N* = 61)		
			Pre-course	Post-course	*p*[†]			Pre-course	Post-course	*p*[‡]
Q3[d]	3.48 (±0.09)	3.91 (±0.09)	3.46 (±0.13)	3.90 (±0.12)	0.001	51.9% (68)	76.0% (95)	54.1% (33)	75.4% (46)	0.009
Q6[e]	3.58 (±0.09)	4.16 (±0.07)	3.66 (±0.12)	4.33 (±0.07)	<0.001	59.5% (78)	92.0% (115)	60.7% (37)	95.1% (58)	<0.001
Q13[f]	4.02 (±0.10)	4.06 (±0.11)	3.92 (±0.15)	4.26 (±0.14)	0.041	82.4% (108)	82.4% (103)	80.3% (49)	85.2% (52)	0.55

[a] *Total pre-course responses (n = 131) ignoring ID code pairing.*
[b] *Total post-course responses (n = 125) ignoring ID code pairing.*
[c] *All pre and post-course responses that were able to be paired by ID codes written by respondents on both pre-course and post-course surveys (n = 61).*
[d] *It is important to have an animal welfare and ethics course as part of the veterinary curriculum.*
[e] *I feel confident that I know how to research an animal welfare topic, even one that I know very little about, in order to form an educated opinion that I can communicate to others.*
[f] *As an expert in a particular animal type, I am obligated to be an advocate for the welfare of all animals in my community.*
[*] *1 = strongly disagree; 5 = strongly agree.*
[†] *Paired t-test.*
‡ *McNemar's test.*

future practice interests, with multiple selections allowed, 52.7% ($n = 69$) of respondents expressed interest in companion animal practice, 26.0% ($n = 34$) were interested in mixed practice, and 20.6% ($n = 27$) were interested in research or academia. A smaller percentage of respondents were interested in exotics or zoo medicine ($n = 17$; 13.0%), equine ($n = 15$; 11.5%), food animal ($n = 12$; 9.2%), public health or policy ($n = 9$; 6.9%), or wildlife ($n = 8$; 6.1%). Practicing lab animal medicine was of least interest to respondents, of which only 2.3% ($n = 3$) expressed interest.

Summary of Responses to Questions Containing Likert Scales or Ranking

Four questions containing either Likert scale responses or ranking responses were selected for analysis from both the pre-course and post-course surveys. These questions are described in **Table 2**. For the first question (Q3), which asked respondents to indicate their level of agreement with the inclusion of an animal welfare and ethics course in the veterinary curriculum, percentages of responses that contained a Likert value >3, indicating agreement with the statement within the question, were calculated. For the paired response data ($n = 61$), a significant increase in the percentage of respondents indicating they agreed with the inclusion of the course was seen in the post-course responses ($p = 0.009$). Before completing the course, 54.1% ($n = 33$) expressed agreement with the inclusion of a course while 75.4% ($n = 46$) expressed agreement after completing the course (**Table 3**).

For Question 6, which asked respondents to indicate, using a Likert scale, their level of agreement with the statement "I feel confident that I know how to research an animal welfare topic, even one that I know very little about, in order to form an educated opinion that I can communicate to others," a significant change in agreement was seen in the paired post-course survey data ($p < 0.001$; **Table 3**); 60.7% ($n = 37$) of respondents expressed agreement with the statement in Q6 before completing

TABLE 4 | Pre-course[a], post-course[b] and paired survey[c] responses to the following question: Q11: How influential should the listed individuals be in making animal welfare decisions within a community?

	Veterinarians					Animal scientists					Animal industry members				
	Pre-course *N* = 110	Post-course *N* = 114	Paired *N* = 47			Pre-course *N* = 110	Post-course *N* = 114	Paired *N* = 47			Pre-course *N* = 110	Post-course *N* = 114	Paired *N* = 47		
			Pre-course	Post-course	*p*[†]			Pre-course	Post-course	*p*[†]			Pre-course	Post-course	*p*[†]
OVERALL MEAN RANK VALUE* (± SEM)	1.31 (±0.06)	1.45 (±0.07)	1.55 (±0.13)	1.28 (±0.08)	0.06	2.04 (±0.07)	1.99 (±0.09)	1.87 (±0.12)	2.02 (±0.12)	0.34	3.29 (±0.10)	3.06 (±0.09)	3.32 (±0.17)	3.17 (±0.12)	0.38
	General public					Animal rights activists/campaigners					Politicians				
	Pre-course *N* = 110	Post-course *N* = 114	Paired *N* = 47			Pre-course *N* = 110	Post-course *N* = 114	Paired *N* = 47			Pre-course *N* = 110	Post-course *N* = 114	Paired *N* = 47		
			Pre-course	Post-course	*p*[†]			Pre-course	Post-course	*p*[†]			Pre-course	Post-course	*p*[†]
OVERALL MEAN RANK VALUE* (± SEM)	4.33 (±0.10)	4.37 (±0.10)	4.40 (±0.17)	4.29 (±0.16)	0.63	4.75 (±0.09)	4.75 (±0.09)	4.66 (±0.12)	4.74 (±0.14)	0.51	5.29 (±0.09)	5.38 (±0.07)	5.19 (±0.16)	5.49 (±0.09)	0.11

[a] *Total pre-course responses (n = 131) ignoring ID code pairing.*
[b] *Total post-course responses (n = 125) ignoring ID code pairing.*
[c] *All pre and post-course responses that were able to be paired by ID codes written by respondents on both pre-course and post-course surveys (n = 61).*
**1 = Highly Influential; 5 = Minimally or not at all influential.*
[†] *Paired T-test.*

the course while 95.1% ($n = 58$) expressed agreement after completing the course. No significant changes in agreement were seen between the paired pre-course and post-course data in Question 13 ($p = 0.55$), which presented respondents with the statement "As an expert in a particular animal type, I am obligated to be an advocate for the welfare of all animals in my community." The majority of pre- and post-course paired survey responses agreed with this statement ($n = 49$; 80.3% and $n = 52$; 85.2%, respectively).

Question 11 asked respondents to rank, in terms of influence, their responses to the question "How influential should the listed individuals be in making animal welfare decisions within a community?" Given that some respondents in each pool did not correctly rank the categories amongst the others, these responses were eliminated from this data set. For the pre-course respondents ($n = 110$) and post-course respondents ($n = 114$), and the paired respondent data ($n = 47$), veterinarians were ranked as the most highly influential, with animal scientists ranked second most influential, animal industry members ranked third, the general public ranked fourth, animal rights activists or campaigners ranked fifth, and politicians were ranked as the least influential (**Table 4**). No significant differences in these rankings were seen between the paired pre-course and post-course data for other members of the community ($p \geq 0.11$).

Associations of Respondent Demographics to Pre-course Likert Scale Response Questions

Demographic data was collected only on the pre-course survey. Percentages of total respondents in demographic categories of gender, home community, and track that responded with Likert values >3 (indicating agreement with the question's statement) were calculated and the paired response data were tested for significant differences by the Fisher Exact method (**Table 5**). There were no significant differences between responses of different genders, home communities, or track selections to questions relating to the inclusion of the welfare course in the veterinary curriculum, respondent confidence in researching welfare issues, or respondent obligation to animal welfare advocacy ($p > 0.05$).

Qualitative Analysis

Five themes were used to code the write-in responses: knowledge and confidence, a sense of duty or responsibility, anger or resentment, seeking change, and undecided. A majority of write-in responses in both the total pre-(62.4%) and total post-course (64.5%) surveys reflected overall positivity toward the new course. Of the five themes used to code the write-in responses to the statement: "It is important to have an animal welfare and ethics course as part of the veterinary curriculum," the theme that occurred in the greatest proportion of paired pre-course and post-course responses was a "Sense of Duty or Responsibility" (43.3% pre-course and 50.0% post-course; **Table 5**). This theme primarily included write-in responses that reflected a strong sense of moral and professional responsibility toward society as a whole ("Veterinarians are expected to have

an opinion on animal welfare"; "Veterinarians pass on education to the public"; "Veterinarians are publically held accountable as stewards of animal welfare"). Responses discussed the necessity of integrity, intelligence, and honesty in a culture that places high value in transparency and dependability. For the paired pre-course responses, the theme of "Anger or Resentment" was the second most common with 40.0% ($n = 12$) prevalence. This theme included write-in responses that reflected negative feelings toward the new course itself, with comments that reflected a general fear of the potential increase in workload ("waste of time.... [third-year students] have too much to learn already."), disinterest in instruction on a topic they believed to be subjective ("[we can] figure this out on [their] own" and "we are becoming vets so we already care about welfare...I don't need someone telling me how best to do that"), and a perceived redundancy in the curriculum ("[we] get welfare throughout"). The proportion of responses with the "Anger or Resentment" theme was reduced by half in the post-course paired responses ($n = 6$; 20.0%). The proportion of paired responses with the theme of "Knowledge and Confidence" did not change considerably from pre-course to post-course ($n = 5$; 16.7% and $n = 6$; 20.0%, respectively). This theme included responses that reflected an overall appreciation for the information that can be gained from the welfare course and responses that discussed the course material's applicability to the veterinary profession. The least common themes for the pre-course responses were "Undecided" ($n = 2$; 6.7%), a theme that included responses that indicated the respondent was "not sure how this class will go," and "Seeking Change" ($n = 0$; 0.0%). The theme of "Seeking Change" included responses in which the respondent made suggestions for how the course or the instruction on welfare could be changed ("An important topic but probably doesn't need a full course"; "[This topic] should be input throughout the curriculum instead"). The theme of "Seeking Change" increased in the post-course paired responses to 16.2% ($n = 5$).

Any differences, or changes, in theme proportions between the total pre-course and post-course response groups cannot accurately be discussed given that more than half of the post-course responses could not be matched with pre-course survey ID codes. However, similar proportions were seen in the total pre-course and post-course data as were seen in the paired response data for the themes of "Undecided," "Sense of Duty or Responsibility," and "Anger or Resentment" (**Table 6**).

DISCUSSION

The intent of this survey was four-fold: (1) to examine the current opinions of third-year veterinary students, never before exposed to a professional degree animal welfare course, toward the implementation of such a required course; (2) to assess these veterinary students' opinions toward their roles within a community in making animal welfare decisions; (3) to assess the confidences of these veterinary students in educating themselves about animal welfare issues; (4) to assess the effect of this new course on changing students' knowledge of and opinions toward their roles as animal welfare advocates. Amidst the growing concern in the veterinary and academic communities regarding the lack of adherence to animal welfare standards and practices by veterinarians (1, 12), it is important to assess what may be missing in veterinary education that could be causing veterinarians to either overlook or not fully understand welfare violations. Veterinarians are not only expected, but obligated to protect, improve, and advocate for animal welfare, regardless of their species specialty. A joint statement by the AVMA, the Federation of Veterinarians of Europe, and the Canadian Veterinary Medical Association states that veterinarians,

"as knowledgeable and accountable professionals—have an opportunity and an obligation to help animal owners, caretakers, handlers, and policy makers protect and improve animals' welfare...veterinarians are, and must continually strive to be, the leading advocates for the good welfare of animals in a continually evolving society" (7).

As society changes and relationships with animals change, veterinarians must remain the constant advocate for animal welfare over the course of animals' lives and for the proper humane treatment at the end of animals' lives.

This study's gender representation is similar to that of the gender composition of veterinary schools nationwide. The 2017–2018 Internal Association of American Veterinary Medical Colleges (AAVMC) Annual Data Report cites that nationwide, approximately 19% of veterinary students are male and, according to the Internal Data Report, approximately 16% of Colorado State University veterinary students are male (20). In terms of home community, the 2017–2018 Internal AAVMC report cites that, nationwide, approximately 55% of applicants identify their home communities as suburban, which is similar to the home community representation in this study (20). Additionally, the demographics is this study were similar to that of other study populations that administered surveys focused on veterinary animal welfare knowledge and education [14, 15].

To the authors' knowledge, there have not been any papers published with data reflecting the relationships of the demographic categories to respondent attitudes toward an animal welfare course. In past survey studies (13, 16), male respondents rated lower than females in their empathy and general attitudes toward animals. In addition, another study found that veterinary students interested in working with small animals rated procedures such as banding castration and castration before 1 week of age as more inhumane than students interested in working with food animals (14). In the current study, there was no significant difference in opinion toward the inclusion of the welfare course between large, small, and general animal track respondents or different genders.

The results of this survey indicate that the course had a positive effect on respondents' opinions toward the inclusion of an animal welfare course in the veterinary curriculum. These results are similar to the post-course sentiments of students at other veterinary schools who agreed that an animal welfare course was "challenging and effective" (17) and should be a vital element of the veterinary curriculum (15, 17). The material presented in the course at CSU may have been more intellectually and emotionally engaging than students had expected. Some respondents may have begun the course wanting

TABLE 5 | Associations of respondent gender, home community, and track selection with pre-course survey responses[a] to questions containing Likert scale responses.

	Q3: It is important to have an animal welfare and ethics course as part of the veterinary curriculum		Q6: I feel confident that I know how to research an animal welfare topic, even one that I know very little about, in order to form an educated opinion that I can communicate to others		Q13: As an expert in a particular animal type, I am obligated to be an advocate for the welfare of all animals in my community	
	**Proportion of respondents with Likert values > 3 (*n*)**	***p****	**Proportion of respondents with Likert values > 3 (*n*)**	***p****	**Proportion of respondents with Likert values > 3 (*n*)**	***p****
**GENDER**						
Male (*n* = 21)	57.1% (12)	0.64	47.6% (10)	0.27	81.0% (17)	0.76
Female (*n* = 110)	50.9% (56)		61.8% (68)		82.7% (91)	
**HOME COMMUNITY**						
Rural (*n* = 30)	46.7% (14)	0.54	66.7% (20)	0.4	70.0% (21)	0.06
Non-rural (*n* = 101)	53.5% (54)		57.4% (58)		86.1% (87)	
**TRACK**						
Small animal (*n* = 71)	49.3% (35)		62.0% (44)		80.3% (57)	
Large animal (*n* = 25)	48.0% (12)	0.53	68.0% (17)	0.27	76.0% (19)	0.21
General (*n* = 35)	60.0% (21)		48.6% (17)		91.4% (32)	

[a] *Total pre-course responses (n = 131) ignoring ID code pairing.*
** Fisher exact test.*

to be convinced of its worth before deciding whether or not they agreed that such a course was necessary, and in the end, were adequately convinced.

Additionally, respondents were asked to provide reasons for their agreement or disagreement with the inclusion of an animal welfare course in the veterinary curriculum. A majority of write-in responses in both the total pre- (62.4%) and total post-course (64.5%) surveys reflected overall positivity toward the new course. Students expressed interest in, and passion for, the topic of animal welfare given their commitment to the professional oath and expectations of the veterinary profession, welfare advocacy in their communities, and their clients and patients. Several of the respondents wrote statements similar to "Veterinarians are publicly held accountable as stewards of animal welfare and we should be knowledgeable on the topic." Through their written comments students conveyed the necessity of understanding animal welfare beyond animal health, and the importance of effectively communicating this knowledge to non-scientific members of their communities.

When considering the post-course percentage of students who agreed that a welfare course should be part of the veterinary curriculum, approximately a quarter of the student respondents did not believe a welfare course should be included in veterinary programs. The question did not specify that the welfare course would be required or elective, simply that a course centered on the topic would be part of the general curriculum. Yet, it is possible that students assumed this question referred to a required curricular element given that they themselves were enrolled in a required course.

Before respondents had taken the course, the proportion of paired write-in responses that reflected anger or resentment about the course was almost equal to the proportion of paired responses that reflected positive sentiments. Within this theme of anger and resentment were responses that discussed the existing heavy third-year course load and that the welfare course would be a "waste of time.... [third-year students] have too much to learn already." Comments such as these reflecting student fears of an increased workload and unmanageable stress create concerns for those constructing veterinary curricula nationwide (10, 13, 21). When introducing a new course into an already full, challenging, and fairly stable veterinary curriculum, finding space in which to fit this new course without sacrificing the time spent in other courses is a real challenge. In this case, students may have felt overwhelmed with the prospect of another course added as they approached their final year.

Another common response within the theme of anger and resentment was that respondents felt they could "figure this out on [their] own." It may be a common response by members of the veterinary community to treat animal welfare as a basic concept that should be second nature to everyone. To be sure, animal welfare should be recognizable to everyone, in particular animal care professionals, yet there often exists a lack of appreciation for some basic welfare principles (1, 12). In a survey completed in the UK in 2000, a proportion of male veterinary students were found to disagree with the notion that cattle and cats can feel pain while also exhibiting a significant decline in empathy toward animals as they progressed through veterinary school (13). In addition, fewer than 90% of veterinary students surveyed in 2005 believed in the cognitive abilities of farm animals whereas over 90% believed dogs and cats were cognitive beings (14). To have knowledge of animal welfare issues demands continuous education and awareness of contemporary issues, such as the recognition and treatment of animal pain, in

TABLE 6 | Qualitative thematic proportions of responses for write-in survey data.

Theme	Example response from data	Average proportion of paired responses pre-course* (*n*) (*n* = 30)	Average proportion of paired responses post-course* (*n*) (*n* = 30)	Average proportion of total pre-course responses* (*n*) (*n* = 93)	Average proportion of total post-course responses* (*n*) (*n* = 79)
Sense of duty or responsibility	"Much of our oath necessitates the commitment to animal welfare and it is a part of our duty to study it."	43.3% (13)	50.0% (15)	47.3% (44)	50.5% (47)
Anger or resentment	"I don't need someone telling me how best to help animals."; "adding to our course-load is not appreciated."	40.0% (12)	20.0% (6)	34.4% (32)	15.1% (14)
Knowledge and confidence	"Animal welfare and ethics may not be obvious to everyone."; "it helps small animal people learn about large animal welfare and vice versa."	16.7% (5)	20.0% (6)	15.1% (14)	14.0% (13)
Undecided	"Not sure what the value of the course will be."; "we'll see."	6.7% (2)	0.0% (0)	5.4% (5)	0.0% (0)
Seeking change	"This would be more beneficial earlier in curriculum or should be optional."	0.0% (0)	16.7% (5)	6.5% (6)	9.7% (9)

**Averaged across both thematic scorers' total proportions for paired response data.*

addition to one's own ideologies. The intent of the course was to provide basic knowledge of welfare issues for all students entering their final year of training and to encourage them to continue their education and consideration of animal welfare issues beyond graduation.

Another common survey response to the inclusion of the welfare course was that respondents "get welfare throughout" the veterinary curriculum. These survey responses indicate that students unhappy with the inclusion of the new welfare course felt confident in their knowledge of animal welfare from exposure to the subject in previous semesters. While it is encouraging that other courses incorporate welfare training, this training was not comprehensive. Ideally, these individuals, upon completion of the course, acknowledged learning new information while having sharpened their existing skills. Additionally, a few respondents wrote sentiments similar to "we are becoming vets so we already care about welfare…I don't need someone telling me how best to do that." This statement makes an incorrect assumption that those interested in animal health and medicine are also proficient in recognizing and addressing poor welfare situations that may not immediately present a physically sick animal. The science of animal welfare includes more than just the assessment of physical aspects of an animal's welfare (i.e., health) and includes non-physical components. When welfare is approached from a purely health perspective, there is a risk that poor welfare may be overlooked. Animal welfare science takes the idea of an animal's well-being beyond humanity's anthropomorphic ideals of what animal happiness and comfort look like and broadens the definition of welfare to a state of existence that is more than just the absence of suffering (22, 23). Despite some of the negative comments, it is encouraging that the prevalence of the theme of "anger and resentment" was reduced by 50% post-course. This indicates that overall, students found the course to be more useful and important than they had previously stated.

The theme of "knowledge and confidence" was less prevalent than expected but the responses helped illustrate that some students felt their welfare education had been insufficient up until the introduction of this course. One respondent wrote "We have not been taught about animal welfare so far and I think it is something that should be part of our curriculum." Although some students had indicated they receive welfare training throughout the curriculum this contrary comment may indicate that not all students have the same perception of what adequate training should include and would like to see more specific inclusion of animal welfare topics. In addition, a few responses indicated that they were concerned with the lack of mutual understanding of the topic of animal welfare amongst their peers, writing that "animal welfare may not be obvious to everyone…it helps small animal people learn about large animal welfare" and vice versa. Some proponents of adding specific welfare courses to veterinary curricula have suggested that without a specific course in animal welfare, it is impossible to know that every veterinary graduate has

a common understanding and respect for animal welfare issues (12, 21).

Finally, the theme of "seeking change" was not seen in pre-course responses but was seen in post-course responses. Perhaps those that felt undecided about the inclusion of this course concluded that they supported the general principles presented but that the method of instruction or the placement within the third-year curriculum could be improved. The main sentiment reflected in responses identified with this theme was that the placement of this welfare course in the second semester of the third year was not ideal. Some respondents suggested the course should be included earlier in the veterinary curriculum, when some necessary foundations of veterinary medicine, like animal welfare, should begin to be established. These sentiments were considered and after internal curricular review it was decided to offer the CSU animal welfare in the first semester of the second year beginning in the 2018–2019 academic year.

The results of this study found a significant improvement in respondents' abilities to research an animal welfare topic with which they were previously unfamiliar. A previous paper discussed a similar finding which highlighted the ability of limited exposure to animal welfare assessment to make an impact on veterinary students' abilities to educate themselves (15). The course likely exposed students to welfare topics they had not explored before and possibly peaked their interest, encouraging them to investigate topics further outside of class and expanding their research skills. By having the ability and confidence to find reliable and unbiased information on animal welfare issues, students are more likely to become veterinarians who will educate their clients and the public in efforts to provide accurate information, and reduce the spread of false information, relating to animal well-being (1, 15, 24).

Interestingly, the course did not have a significant effect on the commitment of veterinary students to advocate for the welfare of all animals in their communities. Both before and after the course, more than 80% of respondents agreed with the obligation, as veterinarians, to act as advocates for all animals' welfare. However, the remaining 15–20% of respondents did not agree with this obligation. It is possible that respondents may have been hesitant to agree with a statement regarding commitment to welfare advocacy that was as bold as to include "*all* animals in [the respondent's] community". This survey statement may have been written too boldly and may have benefited from eliminating the word "all." The intention of this question's statement was to emphasize the all-encompassing, unbiased, and non-specific nature of a veterinarians obligation toward animal welfare. The AVMA states that "veterinarians are obligated morally, ethically, and philosophically to promote the welfare of animals" (14, 25). This statement does not suggest that veterinarians are only obligated to care for the welfare of some animals and disregard that of others. There is concern that veterinary students and practicing veterinarians do not fully understand or appreciate the extent of their roles as animal welfare advocates within their own communities (1, 12, 24). Given the acknowledgment by the authors of this paper that this question's wording may have been too bold and therefore caused unintended effects on the responses, conclusions must be made carefully. However, responses to this question do suggest that there exists a proportion of veterinary students, nearing graduation, that do not believe they are obligated to advocate for all animal well-being. It was not possible with this study to delve deeper into what motivated these responses but would be interesting to include in future work.

When asked to rank members of society in terms of how influential they should be in animal welfare decisions within a community, all respondents ranked veterinarians as ideally having the most influence. Veterinarians hold a special role in society as animal experts with an obligation to both human and animal (1, 12, 24). Veterinarians have a "certified expertise" that comes from both their education and their professional mandate (24) and society expects them to wield this professional status to answer questions, find solutions, and prevent future problems between animals and humans (and at times, animals and animals). After completing the welfare course, respondent opinion toward the role of veterinarians in society did not change significantly. These findings are in contrast to another study which found that a lower percentage of course participants, after completing the course, ranked veterinarians and members of the AVMA as important in animal-welfare decision making, compared to non-course participants (15). Lord et al. (15) suggested that, after completing the course, respondents realized the importance of the public and the constraints of science in influencing animal welfare decisions within the community and within animal industries. In the current study, respondent opinion toward the importance of the general public in influencing animal welfare decisions was not changed post-course. It is possible that the focus of this specific course was more encouraging of the veterinarian as a major influence within society as a means of encouraging the students to become engaged in the topic of animal welfare.

The introduction of an animal welfare course in the third-year veterinary curriculum at CSU demonstrated improvements in student understanding of the value of animal welfare science education and in their ability to conduct research and self-educate. Specific sentiments regarding the introduction of an animal welfare course into the veterinary curriculum were highlighted in a qualitative analysis that exposed and quantified both positive and negative attitudes toward focused welfare education. The value students placed on the influential role of veterinarians with respect to animal welfare issues in their communities was held high.

Future research should be performed on a national scale investigating the potential gaps in veterinary education pertaining to animal welfare that may exist in order to further encourage the development of mandatory animal welfare science courses in veterinary curriculums nationwide. Research should also be conducted to better understand veterinary students' opinions of how their roles as animal welfare advocates could be better supported in their education and how this advocacy could be better executed within their communities.

ETHICS STATEMENT

The study was submitted for full review to Colorado State University's Institutional Review Board. After initial review it was exempt from full review due to its limited risk to participants.

AUTHOR CONTRIBUTIONS

EJ, MF, LL, AB, and LE-C have substantial contribution to the work through concept, design, analysis and/or interpretation. Additionally, all authors revised and edited the draft and approve of publication.

REFERENCES

1. Hewson CJ. Focus on animal welfare. *CVJ*. (2003) 44:335–6.
2. Estol LR. Animal welfare in the veterinary curriculum. In: *Proceedings of: Global Conference on Animal Welfare: An OIE Initiative*. Paris: European Communities (2004). p. 33–45.
3. Broom DM. Animal welfare education: development and prospects. *J Vet Med Educ*. (2005) 32:438–44. doi: 10.3138/jvme.32.4.438
4. WSAVA (World Small Animal Veterinary Association). *International Veterinary Oaths*. (2018). Available online at: https://www.wsava.org/WSAVA/media/PDF_old/Global-Veterinary-Oath.pdf (accessed January 10, 2019).
5. Broom DM. Animal welfare: an aspect of care, sustainability, and food quality required by the public. *J Vet Med Educ.* (2010) 37:83–8. doi: 10.3138/jvme.37.1.83
6. Thiermann A, Babcock S. Animal welfare and international trade. *Rev Sci Tech.* (2005) 24:747–55. doi: 10.20506/rst.24.2.1600
7. AVMA (American Veterinary Medical Association). *Joint AVMA-FVE-CVMA Statement on the Roles of Veterinarians in Ensuring Good Animal Welfare*. (2018). Available online at: https://www.avma.org/KB/Policies/Pages/Joint-Statement-Animal-Welfare.aspx (accessed June 6, 2018).
8. AVMA (American Veterinary Medical Association). *Veterinarian's Oath*. (2018). Available online at: https://www.avma.org/KB/Policies/Pages/veterinarians-oath.aspx (accessed June 6, 2018).
9. AVMC/AAVMC (American Veterinary Medical Associaiton/American Association of Veterinary Medical Colleges). *Animal Welfare in Veterinary Medical Education and Research. Summary of International Educational Symposium on Animal Welfare*. (2009). Available online at: https://www.avma.org/Events/Symposiums/AnimalWelfare/Pages/default.aspx (accessed January 10, 2019).
10. Shivley CB, Garry FB, Kogan LR, Grandin T. Survey of animal welfare, animal behavior, and animal ethics courses in the curricula of AVMA Council on Education-accredited veterinary colleges and schools. *J Am Vet Med Assoc*. (2016) 248:1165–70. doi: 10.2460/javma.248.10.1165
11. AVMA (American Veterinary Medical Association). *COE Accredation Policies and Procedures: Requirements*. (2017). Available online at: https://www.avma.org/ProfessionalDevelopment/Education/Accreditation/ Colleges/Page/coe-pp-requirements-of-accredited-college.aspx (accessed June 6, 2018).
12. Main DC. Evolution of animal-welfare education for veterinary students. *J Vet Med Educ.* (2010) 37:30–5. doi: 10.3138/jvme.37.1.30
13. Paul ES, Podberscek AL. Veterinary education and students' attitudes towards animal welfare. *Vet Rec*. (2000) 146:269–72. doi: 10.1136/vr.146.10.269
14. Levine ED, Mills DS, Houpt KA. Attitudes of veterinary students at one US college toward factors relating to farm animal welfare. *J Vet Med Educ.* (2005) 32:481–90. doi: 10.3138/jvme.32.4.481
15. Lord LK, Walker JB, Croney CC, Golab GC. A comparison of veterinary students enrolled and not enrolled in an animal-welfare course. *J Vet Med Educ*. (2010) 37:40–8. doi: 10.3138/jvme.37.1.40
16. Hazel SJ, Signal TD, Taylor N. Can teaching veterinary and animal-science students about animal welfare affect their attitude toward animals and human-related empathy? *J Vet Med Educ*. (2011) 38:74–83. doi: 10.3138/jvme.38.1.74
17. Abood SK, Siegford JM. Student perceptions of an animal-welfare and ethics course taught early in the veterinary curriculum. *J Vet Med Educ*. (2012) 39:136–41. doi: 10.3138/jvme.0911.093R.1
18. Verrinder JM, Phillips CJ. Assessing veterinary and animal science students' moral judgment development on animal ethics issues. *J Vet Med Educ*. (2015) 42:206–16. doi: 10.3138/jvme.0215-022R
19. Lord LK, Millman ST, Carbone L, Cook N, Fisher A, McKeegan D, et al. A model curriculum for the study of animal welfare in colleges and schools of veterinary medicine. *J Am Vet Med Assoc*. (2017) 250:632–40. doi: 10.2460/javma.250.6.632
20. AAVMC (Association of American Veterinary Medical Colleges). *Annual Data Report 2017–2018*. (2018). Available online at: http://www.aavmc.org/about-aavmc/public-data (accessed July 1, 2018).
21. Lord LK, Walker JB. An approach to teaching animal welfare issues at the Ohio State University. *J Vet Med Educ.* (2009) 36:276–9. doi: 10.3138/jvme.36.3.276
22. Rollin BE. Animal pain. In: Fox MW, Mickley LD, editors. *Advances in Animal Welfare Science.* Dordrecht: Springer (1986). p. 91–106.
23. Rollin BE. Animal pain: what it is and why it matters. *J Ethics*. (2011) 15:425–37. doi: 10.1007/s10892-010-9090-y
24. Carbone L. Expertise and advocacy in animal-welfare decision making: considerations for a veterinary curriculum in animal welfare. *J Vet Med Educ.* (2010) 37:36–9. doi: 10.3138/jvme.37.1.36
25. AVMA (American Veterinary Medical Association). *Animal Welfare Position Statements*. (2018). Available online at: https://www.avma.org/KB/Resources/Reference/AnimalWelfare/Pages/Animal-Welfare-Policy-statements.aspx (accessed May 10, 2018).

Conflict of Interest Statement: The authors declare that the research was conducted in the absence of any commercial or financial relationships that could be construed as a potential conflict of interest.

Copyright © 2019 Johnstone, Frye, Lord, Baysinger and Edwards-Callaway. This is an open-access article distributed under the terms of the Creative Commons Attribution License (CC BY). The use, distribution or reproduction in other forums is permitted, provided the original author(s) and the copyright owner(s) are credited and that the original publication in this journal is cited, in accordance with accepted academic practice. No use, distribution or reproduction is permitted which does not comply with these terms.
