# Supplementary material for: Laboratory animal ethics education improves medical students' awareness of laboratory animal ethics
Source: BMC Med Educ. 2024 Jul 1;24:709. doi: 10.1186/s12909-024-05703-9 (PMC11218205; doi:10.1186/s12909-024-05703-9)
Supplement: Supplementary file 1 — Supplementary Material 1. [file 12909_2024_5703_MOESM1_ESM.zip › The revised table/Knowledge, Attitudes, and Practices on Ethics in Biomedical Animal Research in Mexico.pdf]

Journal of the American Association for Laboratory Animal Science
Vol 62, No 6
November 2023
Pages 478–486
Copyright 2023
by the American Association for Laboratory Animal Science

Knowledge, Attitudes, and Practices on Ethics in Biomedical Animal Research in Mexico

Anayántzin Heredia-Antúnez,[1,#] Miguel Galarde-López,[2,#] Elizabeth Téllez-Ballesteros,[3] and Beatriz Vanda-Cantón[3,*]

The most widely accepted ethical concept for the mitigation of harm to animals used in biomedical research is known as the 3Rs, which refer to replacement, reduction, and refinement. The aim of our study was to determine the ethical and regulatory criteria that researchers in Mexico consider when developing their animal research protocols and that members of the ethics committees use when they evaluate and approve these protocols. We circulated a survey to 300 individuals from different research institutions and received responses from 179 researchers and members of ethics committees on questions related to their knowledge, attitudes, and practices toward the use of animals in research based on the 3Rs. The responses obtained indicate that the respondents were aware of the 3R concept, and they claim to apply these principles. However, the responses revealed resistance to using alternatives for research, testing, and teaching (66%). Nineteen percent of the researchers reported that their institutions do not have an integrated Institutional Animal Care and Use Committee (IACUC). Around 80% of respondents were aware of Mexican regulations. The knowledge and application of the 3Rs by researchers and members of the IACUC is a fundamental concept in animal research. Such knowledge contributes the use of ethical standards, attitudes, and practices relevant to the use of animals in research.

Abbreviations and Acronyms: Institutional Animal Care and Use Committee (IACUC)

DOI: 10.30802/AALAS-JAALAS-23-000012

Introduction

Animal research has been crucial to understanding biologic processes and disease mechanisms, developing and testing vaccines, and searching for more and better treatments that benefit of humans and other animals.[5,45] According to data from the European Union, more than 11.6 million animals of all types were used for research purposes in 2019, while in the United States of America, approximately 800,000 animals were used for research in the same year. The data are not comparable due to differences in compiling the numbers of animals used, but they nonetheless reflect the numbers of animals used for this purpose.[61] However, these data are underestimates because they do not include mice, rats, fish or birds.[40,55] In 2019, more than two-thirds of the animals were used in basic (45%) and applied (23%) research, while about one-quarter (23%) were used in drug and other chemical testing to meet regulatory requirements. Other forms of animal use include routine production of biologic agents, such as vaccines, and teaching.[53,61]

In general, obtaining an accurate count of the number of animals used in research worldwide is difficult because some countries do not track the number of animals used.[53] Although animals continue to be necessary for advances in biomedical research, animal research must be conducted within an ethical framework.

Submitted: 10 Feb 2023. Revision requested: 23 Mar 2023. Accepted: 06 Jun 2023.
[1]Postgraduate Medical, Dental and Health Sciences, National Autonomous University of Mexico, Mexico City, Mexico; [2]Research Center on Infectious Diseases, National Institute of Public Health, Morelos, Mexico; and [3]Faculty of Veterinary Medicine and Zootechnics, National Autonomous University of Mexico, Mexico City, Mexico
**Corresponding author. Email: daktari@unam.mx*
#These authors contributed equally to this work. The order of co-first authors was determined by discussion and mutual agreement between the two co-first authors.

The 3Rs concepts have become an ethical benchmark for animal research and have been explicitly or implicitly adopted in the legislation of virtually all countries.[46] The 3Rs were originally developed to reduce the harm and negative impact of research procedures on animals.

- *Replacement* consists of replacing the use of animals with alternatives, which can include animal tissues or ethically obtained human tissues, computer models, so-called organs on a chip, and in silico techniques such as computer modeling. Cell culture, microorganisms, and invertebrates could also be used . In practice, replacement is the most difficult of the 3Rs to implement.
- *Reduction* is the use of the smallest number of animals consistent with statistically sound studies and avoidance of unnecessary duplication of experiments.
- *Refinement* is the use of less invasive techniques and procedures, effective use of anesthesia and analgesia to reduce pain, anxiety, and discomfort, and environmental enrichment programs that improve animal welfare from birth to death.[40,41,52]

Mexico has about 60 animal facilities that are registered and authorized by the Health, Safety, and Agrifood Quality Service (SENASICA) of the Ministry of Agriculture (SADER). These facilities animals such as rodents, rabbits, birds, fish, reptiles, pigs, ruminants, horses, and nonhuman primates, among others. Approximately 60% of these are affiliated with Higher Education Institutions (HEI), 35% with private industries, and 5% with national health institutions (Ministry of Health, Mexican Social Security Institute, among others).[14], The Official Mexican Standards, NOM-062-ZOO-1999 “Technical specifications for the production, care, and use of laboratory animals” establish

the conditions that must be met to allow their use. According to the regulation, animals can be used in research only if 1) the research is essential and knowledge cannot be obtained by other means, 2) a substitute for animals in not available (for example, in vitro cultures, simulators, mathematical models), and 3) the results cannot be predicted and similar studies have not been published.[13,16,39]

In some countries, animal experimentation must be authorized by an ethics committee with officially appointed members who evaluate the research protocol from both ethical and methodological perspectives.[29] In Mexico, a protocol must be approved by an IACUC before the research is initiated. If the investigator's institution does not have an IACUC, he/she may request the support of another institution that does have an IACUC to obtain this approval.[13] Ethical evaluation of the use of animals in research involves weighing the harms to animals against the expected benefits, the justification for the use of animals, the importance of the experiments, and the methodology employed.[44] In Mexico, a harm-benefit analysis is not usually included in animal research protocols because it is not required in the regulations. Therefore, the focus is on compliance with the 3Rs. In contrast the European directive requires that animal research protocols be evaluated from the harm-benefit point of view, and the U.S. Guide for the Care and Use of Laboratory Animals requires IACUCs to perform a similar evaluation.[16] The basic ethical principle underlying the regulation of animal experimentation is that the probable benefits to animals or people should outweigh the harm and suffering caused to the experimental animals; therefore, researchers must explicitly clarify the purpose of the study to be performed and explain why it is important.[48,50] The aim of this study was to identify the ethical and regulatory criteria that researchers and members of ethics committees consider when developing and evaluating animal research protocols in Mexico.

Materials and Methods

Design and study population. The cross-sectional descriptive study was conducted by using a survey aimed at researchers and members of Institutional Animal Care and Use Committees (IACUCs) at universities, institutions of the Ministry of Health, and drug testing laboratories in Mexico during the period from 2018 to 2021.

The survey included both researchers and members of ethics committees and was conducted both in person and online (using the Google Forms platform[22]). The virtual approach was necessary due to the SARS-COV-2 social distancing policies that were applied in Mexico. Participants were selected from public and private research institutions (universities, Ministry of Health institutions and pharmaceutical companies) by using the national transparency platform and the National Directory of Animal Facilities (Figure 1).[14,31] The National Transparency Platform (PNT) allows citizens in general to submit requests for public information and for access, rectification, cancellation, opposition or portability of personal data (ARCOP) to the various regulated entities of the three levels of government in Mexico.

The survey contained 28 items, divided into 6 sections: 1) profile of the researchers and/or committee members, 2) opportunity to serve on an IACUC, 3) selection of the animal model used in the research, 4) application of the ethical principles in animal research (3Rs), 5) knowledge of the regulatory framework, and 6) criteria for approving or developing a research protocol. The questions had been validated[19,21] and were modified and piloted for our study.

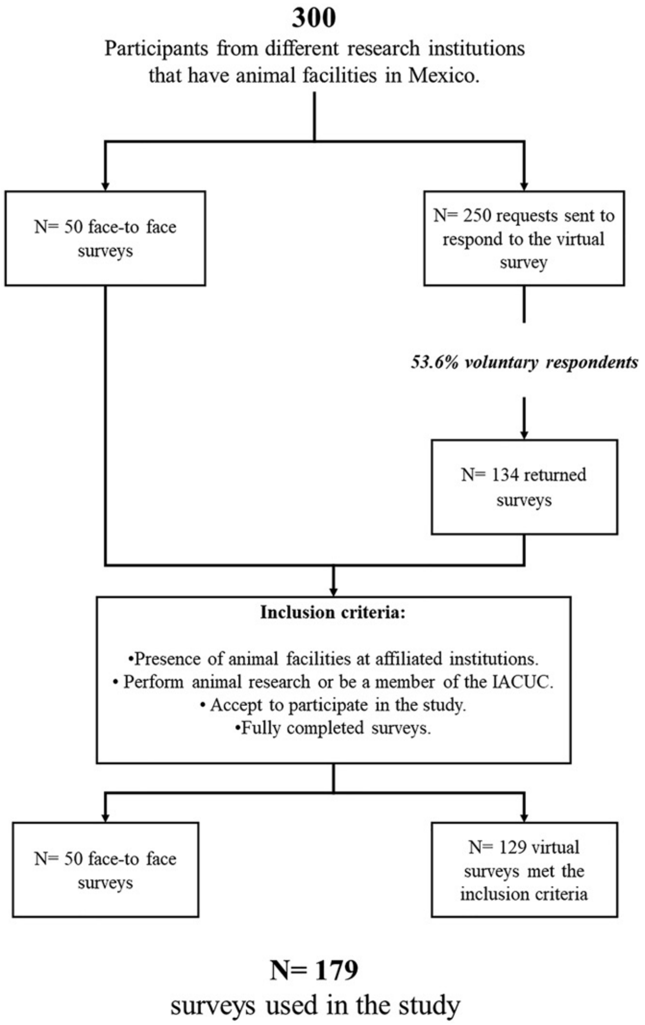

Figure 1. Flow diagram summarizing the survey methods.

Data analysis. We analyzed the variables of interest to obtain measures of central tendency, frequencies and proportions. Only descriptive statistics are provided. Data were analyzed using the statistical program of data science (STATA v.15, Stata Corp, College Station, TX).[51]

Ethical approval statement. The study was approved by the Ethics and Research Committee of the Master and Doctoral Program in Medical, Dental, and Health Sciences, Mexico, Reg. No. PMDCMOS/CEI/005/2019. The confidentiality of the respondents and their institution of affiliation was maintained.

Results

We administered 50 face-to-face surveys and had a response rate of 54% to the 250 mail requests, resulting in 134 returned surveys, 129 of which met the inclusion criteria. All surveyed individuals were researchers in metro Mexico City.

Of the 179 usable surveys (50 face-to-face and 129 virtual). 49% were from women and 51% from men; the mean age was 40 y (sd, 13 y) and ranged from 21 to 90 y. Affiliations were as follows: 51% worked in universities, 27% in hospitals and Ministry of Health institutes, 4% in the pharmaceutical industry, 4% in private industry, and 13% did not indicate the sector to which they belonged. Of the respondents, 88% worked with animals, while 12% had previous experience with animals.

81% reported that their institution had an IACUC. Among the remaining 19% (34) of participants, 39% (13) reported that

creation of an IACUC was in process, 36% (12) did not know why an IACUC was not already present, and 24% gave of reasons why an IACUC was not present (lack of institutional (15%, 5), or faculty (6%, 2) interest, and other reasons (3%, 1)).

Only 15% of the respondents were IACUC members. Among the respondents, 18% had some training in bioethics (courses, certificates, and postgraduate degrees, among others) (Table 1).

Types of research performed with animals. Researchers were asked to indicate areas of research for which they did and did not use animals. The most common areas of animal use included basic research (60%), applied research for human benefit (45%), and for education and training (42%). The most common areas for which animals were never used by the respondents included toxicological research (78%), development, production, control or testing of drugs and/or vaccines (74%,), and disease diagnosis (62%,) and applied research for the benefit of animals (53%,) (Table 2). Percentages reported total over 100% due to multiple responses by individuals to the same question.

Table 1. Characteristics of the study population

Variable	n = 179
Age (y) (Mean, SD, range)	40, 13, (21–90)
Sex	
Female	87 (49)
Male	92 (51)
Respondents	
Use animals in research.	157 (88%)
Do not use animals in research	22 (12%)
IACUC Committee member	
Yes	26 (15%)
No	153 (86%)
Had taken bioethics courses	
Yes	32 (18%)
No	147 (82%)
Institution has an IACUC	
Yes	145 (81)
No	34 (19)
Affiliation	
Academia	92 (51%)
Pharmaceutical industry	7 (4%)
Hospital	49 (27%)
Private company	7 (4%)
No IACUC at Institution (n = 34)	
Lack of institutional interest	5 (15%)
Lack of interest by researchers	2 (6%)
In the process of creating an IACUC	13 (39%)
Did not know the reason	12 (36%)
Another reason	1 (3%)

Alternatives to the use of traditional research species. Respondents were asked to indicate which potential replacements for typical research species were most feasible. These were ranked as follows: in vitro models, 67%; use of invertebrates, 49%; use of animal embryos, 38%; virtual simulations or models, 37%; farm animals, 35%; and biochips or organs on a chip, 32%, and cadavers, 17%).

Ethical attitude toward the animal species used in research. Respondents were asked to score their acceptance to using various animals for research. "Totally acceptable" or "acceptable" choices were animal embryos (100%), pigs (97%), zebrafish (89%), fruit flies (84%), and rats (80%).

The least acceptable ("totally unacceptable" or "unacceptable") were chimpanzees (100%), dogs (51%), and pigs (2%). Complete data are shown in Table 3.

Among the 179 respondents, the reasons for accepting or not accepting the use of certain animal species in the research were as follows: legal restrictions, 59%; degree of sensitivity to pain, 32%; phylogenetic proximity to humans, 27%; and perceived strength of the human-animal bond with that species, 6%.

Application of the 3Rs. Regarding the replacement of research animals, 66% of the respondents agreed that replacement of vertebrate models could not be implemented due to lack of confidence of obtaining equally reliable results. In addition, 20% agreed that information on alternative methods is not easy to find. However, 73% agreed that if the objectives of research protocols could be achieved by using alternative methods, they would apply them. Regarding the reduction of the number of research animals, 94% would agree to perform an extensive literature review to justify their projects and avoid unnecessary repetitions, and 87% claimed to use a statistical justification for the number of animals used in their experiments.

Addressing refinement, 83% agreed that the use of anesthesia and analgesia was indispensable in invasive experiments that cause discomfort and pain to the animals. However, 19% would hesitate to use them if their use was likely to interfere with the results. In addition, 75% agreed that when an

Table 2. Frequency with which animal experiments were performed for the purposes indicated

Purpose n = 179	Never	Almost never	Sometimes	Almost always	Always	Mean attitude score	95% CI
Development, production, control or testing of drugs and/or vaccines	74%	12%	6%	5%	3%	1.5	1.3-1.6
Toxicological research	78%	13%	7%	0.5%	1.7%	1.3	1.2-1.4
Disease diagnosis	62%	11%	22%	2%	3%	1.7	1.5-1.8
Education and training	32%	8%	16%	13%	31%	3.0	2.7-3.2
Basic research	13%	2%	5%	20%	60%	4.1	3.9-4.3
Applied research to benefit of humans	27%	3%	6%	19%	45%	3.5	3.2-3.7
Applied research to benefit of animals	52%	24%	14%	2%	8%	1.9	1.7-2.0

Respondents received a score of 1 for answering "Never"; 2) "Almost never"; 3) "Sometimes"; 4) "Almost always"; 5) "Always". Therefore, a mean score near 5 for each statement indicates a frequent use toward animal species while a score near 1 indicates a disuse. 95% CI, 95% confidence interval.

Table 3. Degree of acceptance or rejection of the use of animals in experiments based on the species

Species	Totally unacceptable	Unacceptable	No opinion	Acceptable	Totally acceptable	Mean attitude score	95% CI
Embryos (different animal species)	0%	0%	0%	0%	100%	5.0	. - .
Dogs	21%	31%	15%	22%	11%	2.7	2.5-2.9
Rats	0%	0%	20%	39%	41%	4.2	4.1-4.3
Fruit Fly (*Drosophila*)	0%	0%	12%	14%	74%	4.6	4.5-4.7
Pigs	1%	1%	0%	37%	61%	4.5	4.4-4.6
Zebrafish	0%	0%	11%	37%	52%	4.4	4.3-4.5
Chimpanzees	19%	81%	0%	0%	0%	1.8	1.7-1.9

Respondents received a score of 1 for answering "Totally unacceptable"; 2) "Unacceptable"; 3) "No opinion"; 4) "Acceptable"; 5) "Totally acceptable". Therefore, a mean score near 5 for each statement indicates a positive attitude toward animal species while a score near 1 indicates a negative attitude. 95% CI, 95% confidence interval.

experimental procedure had a direct negative impact on the health and welfare of the animals, the procedure should be terminated and the animals either euthanized, removed from the study, or given treatment even though the experiment had not been concluded. In the case of choosing a method of euthanasia, 25% thought that selection of the method should not consider cost (Table 4).

Performance of the IACUC. When asked about the authority of the IACUC, 69% agreed that the IACUC should have the authority to stop experiments when animal health and/or welfare would be compromised. Also 62% agreed that compliance with IACUC recommendations should be left to the discretion of the researcher. Regarding the constitution of the IACUC, 85% thought that the IACUC should include only physicians or researchers in the field and 87% felt that members should have additional training in bioethics topics.

Regarding use of the IACUC, 85% thought that a committee member should be consulted during the development of the research protocol to obtain relevant opinions. Finally, 73% strongly agreed that the committee should sanction an investigator

Table 4. Regarding the use of animals for biomedical research

To what extent do you agree with the following statements? (n = 179)	Strongly disagree	Disagree	No opinion	Agree	Strongly agree	Mean attitude score	95% CI
I hesitate to apply any changes in my methodology to substitute vertebrate animals because I am not yet confident of obtaining equally reliable results with other models	1%	7%	26%	37%	29%	3.8	3.7-3.9
Information on alternative methods to animal use in my line of research is easy to find and acquire	12%	21%	47%	17%	3%	2.7	2.6-2.9
In invasive experiments that cause pain or discomfort to the animal, the use of anesthesia and analgesia during and after the procedure is indispensable	1%	0%	16%	20%	63%	4.4	4.3-4.5
Analgesics should be to use in procedures that generate pain in the animals, even if they modify the possible results of their condition	24%	26%	32%	12%	6%	3.4	3.3-3.6
When experimental procedures are performed and the animal presents evident pain and deterioration in its status of health, it should be removed from the group, administered treatment, or euthanized even if the experimental time has not elapsed	1%	3%	21%	16%	59%	4.2	4.1-4.4
If a method of animal death is to be applied, the most economical one should be chosen	25%	24%	26%	9%	16%	2.6	2.4-2.8
If the research objectives can be carried out using cadavers, isolated organs, cell cultures, simulators, or natural animal models, they should be implemented immediately	6%	5%	16%	14%	59%	4.14	3.9-4.3
Before initiating or evaluating a project, a thorough review of the scientific literature should be conducted to obtain original knowledge	0%	0%	5%	13%	82%	4.75	4.6-4.8
To determine the number of animals in the experimental groups, statistical tests including the number of groups, and the variables to be compared should be performed	1%	3%	8%	11%	77%	4.58	4.4-4.7

Respondents received a score of 1 for answering "Strongly disagree"; 2) "Disagree"; 3) "No opinion"; 4) "Agree"; 5) "Strongly agree". Therefore, a mean score near 5 for each statement indicates a positive attitude toward animal research while a score near 1 indicates a negative attitude. 95% CI, 95% confidence interval.

Table 5. Opinions on IACUC functions

To what extent do you agree with the following statements? (n = 179)	Strongly disagree	Disagree	No opinion	Agree	Strongly agree	Mean attitude score	95% CI
The IACUC must stop experiments when animal welfare is compromised during the conduct of research	2%	4%	24%	29%	41%	4.0	3.8-4.1
The decision to implement the recommendations an IACUC should be left to the discretion of the researcher	4%	11%	23%	32%	30%	3.7	3.5-3.9
IACUCs are not required to evaluate protocols involving the use of animals	51%	33%	10%	2%	4%	4.2	4.0-4.4
The desirable profile of an IACUC member involved in the review of protocols using animals should include physicians or researchers in the field	3%	1%	12%	30%	55%	4.3	4.2-4.4
An IACUC member involved in the development or review of protocols involving the use of animals must be trained in ethics or bioethics	2%	1%	9%	38%	50%	4.3	4.1-4.4
An appropriate sanction for researchers who do not follow the IACUC recommendations should be suspension of the experiment	0%	10%	18%	42%	30%	3.9	3.7-4.0
To obtain prompt and relevant opinions, a member of the IACUC should be consulted during the development of the research protocol	0%	0%	15%	37%	48%	4.3	4.2-4.4

Respondents received a score of 1 for answering “Strongly disagree”; 2) “Disagree”; 3) “No opinion”; 4) “Agree”; 5) “Strongly agree”. Therefore, a mean score near 5 for each statement indicates a positive attitude toward animal research while a score near 1 indicates a negative attitude. 95% CI, 95% confidence interval.

or stop an experiment if it did not adhere to the requirements issued by the IACUC (Table 5).

Regulations. Regarding the guidelines, documents, or regulations consulted to develop or evaluate a research protocol involving the use of animals, 81% of respondents referred to NOM-062-ZOO-1999 in an open-ended response (although not by name),17% did not know about a reference document, and 2% mentioned the US Guide for the Care and Use of Laboratory Animals.[12]

Main aspects considered in the development and approval of research protocols involving animals. Respondents were asked to rate the importance of 17 elements in an animal protocol (Table X). In the order of most important to the least important, items were ranked from the highest (92%) to the lowest (21%) as follows: selection of the animal model, scientific validity, the number of animals used, the duration of the experiment, routes, frequency, and volume of sampling, use of environmental enrichment programs, degree of invasiveness, risk to humans, use of anesthetics and analgesics, and methods of euthanasia (Figure 2).

Discussion

Of the total (n = 179) number of the surveys answered, almost 50% of the respondents worked at a higher education institution, 27% at hospitals, and a smaller number at private institutions. This distribution was probably related to the fact that the greatest proportion of respondents were from Mexico City. According to the National Directory of Animal Facilities for 2020, at a national level, 40% are in a city, as are the greatest number of universities and institutions in the health sector.[14] Because we wanted to maintain confidentiality for the respondents, we did not determine whether respondents were from the same institution, including ours, or whether respondents from the same institution had similar responses.

Only 18% of the respondents had taken bioethics courses, even though 87% use animals in their research, indicating a low level of training in bioethics and animal welfare in research. Individuals involved in the development or evaluation of experimental protocols that use animals should consider their management and care, as well as legal considerations and the bioethical principles oriented to the 3Rs.[18,30] This knowledge is important for the members of committees that review animal research protocols and should, include an evaluation of harm-benefit and ethical issues.[6,24,26]

Around 20% of the institutions that use animals for research, experimentation, and/or teaching do not have an IACUC despite the fact that the Official Mexican Standards NOM-062-ZOO-1999 “Technical specifications for the production, care, and use of laboratory animals”, indicates that all institutions that conduct scientific research, technological development, experimentation, and teaching must form an IACUC.[13] In addition, Article 47 of the Mexico City Animal Protection Law establishes that animal experiments shall be carried out in accordance with official Mexican standards and fully justified before institutional bioethics committees.[20] However, none of the existing Mexican regulatory documents oblige institutions with animal facility or that use animals in research to register an IACUC, so no standard IACUC guidelines are available in Mexico. The regulatory documents do not discuss IACUC roles, functions, or reporting requirements, and they also do not include reporting requirements for the number and type of animals used. Accordingly, the use of animals in research is under-reported in Mexico.[27]

Although the fundamental principles of the Basel Declaration[62] recognize that basic research cannot be separated from applied research, the main use of animals by respondents 80% for basic research, 64% for applied research for humans, and 11% for applied research for animals. This result is consistent with the fact that Mexico offers more funding for

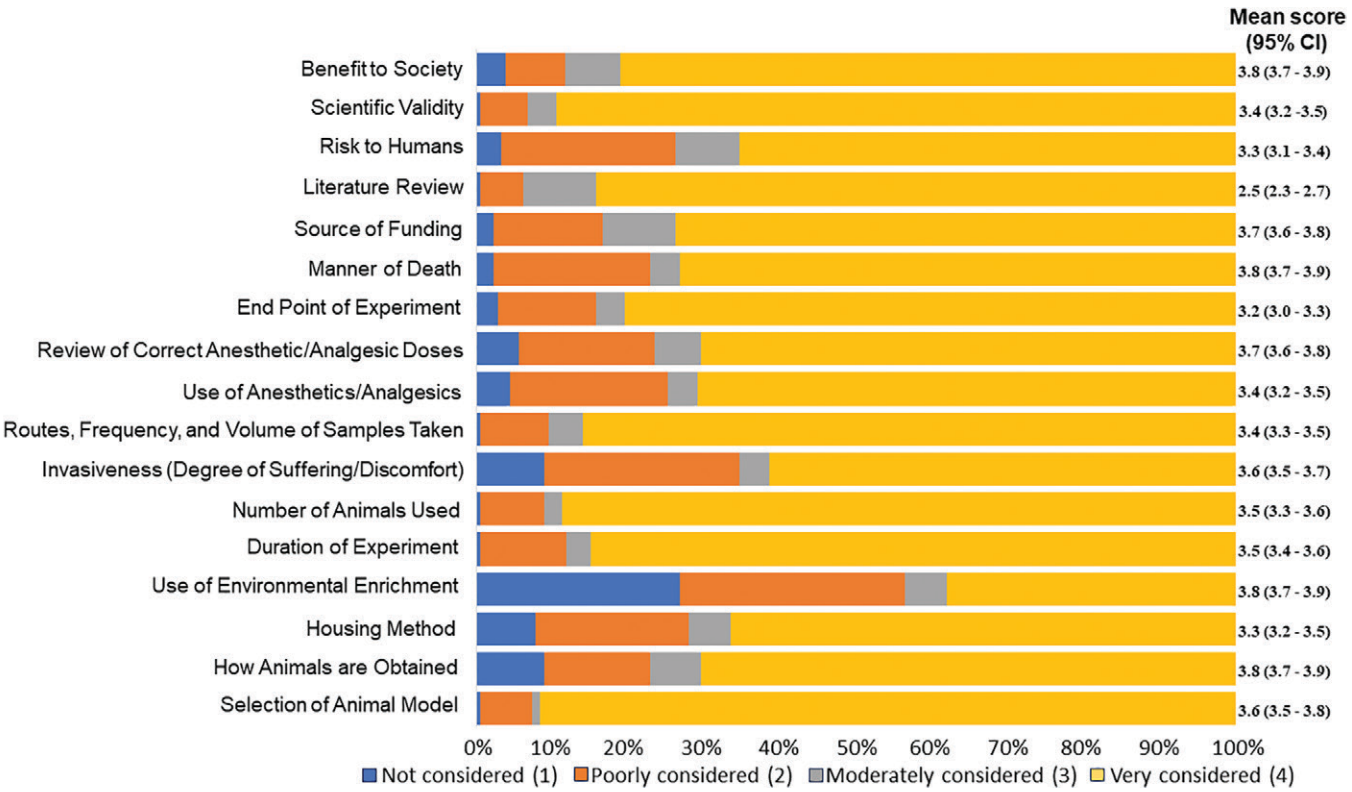

Figure 2. Factors in the development and evaluation of research protocols using animals. A mean score close to 4 for each statement indicates a factor considered in the development and evaluation of animal research protocols, whereas a score close to 1 indicates a factor not considered. CI 95: Confidence Interval to 95%.

basic research projects, which may or may not culminate in applied knowledge.[37] Given that one of the rationales for the use of animal models is cross-species similarity, more studies that benefit nonhuman animals should be encouraged.[4,59] Our study did not evaluate differences in perceptions about animal-based research of individuals who do and do not use animals in research.

With regard to using animals for teaching to assist in the acquisition of professional competencies in the biomedical area, it is not clear why so many animals are still used, even though multiple alternative methods have been developed to both replace and reduce the number of animals in order to apply the principle of the 3Rs in teaching.[47,56] One groups[60] found a strong belief that a living being must be used for optimal learning, and no alternative models adequately simulate such experience. Better education and training of instructors are necessary to prompt more use of non-animal alternatives to achieve learning objectives.

Regarding the alternative methods used in animal research, 94% of the respondents were aware of alternative methods, with cell culture being the most reported. Respondents generally had little confidence in the other alternatives listed and claimed that information about alternatives was hard to find.[11,32,42,43] Although some alternatives present economic or technical limitations,[17] institutions appear to have little interest in promoting the generation of alternatives to animal use that can meet their needs in each context. Researchers should show greater interest in applying or developing alternatives through multidisciplinary work.

Based on the ethical issues raised using animals in current research, the results showed a preference for the use of animal embryonic and invertebrate models, although how the embryos were obtained, and the relevant species were not considered. This result is not unexpected due to the incomplete neurological development and nociception of embryos.[25] and a general lack of concern regarding nociception and sensation mechanisms in invertebrates such as flies.[7,35,49] Historically, laws, ethical codes and guidelines have recommended the use of invertebrates as partial surrogates for vertebrates.[15,33,39] On the other hand, our respondents had less concern for vertebrates such as pigs, zebrafish, and rats; preferred species have clear advantages in terms of their management, husbandry, and widespread use in research. Others[57] have noted that the selection of an animal model is heavily weighted to the preferences of a researcher based on their personal experience or on the availability of the species at their institution, rather than on a careful query into the most appropriate model for their research question. The human relationship with an animal species was the least important consideration of respondents in the development of a research protocol, but the dog was the species that most considered totally unacceptable for research. This perception may have been influenced by the legal restrictions established by the Mexico City Animal Protection Law (Art. 49), which prohibits the use of dogs and cats for research and teaching purposes.[20]

Regarding knowledge of the implementation and application of the 3Rs in animal research protocols, 66% of the respondents doubted whether alternative methods would help them obtain data as dependable as that obtained by using with animals, but if they were certain that they could achieve their objectives by using alternative methods, they would use them. Perhaps, the implementation of institutional training programs and workshops on the search for alternative methods[23] would be helpful to bring more awareness about the usefulness of alternative

methods to animal research. A high proportion of respondents recognize the importance of the literature review as part of the ethical commitment of the researcher to avoid unnecessary duplication (94%).[36]

Although 87% of respondents reported having performed statistical tests to justify the number of animals used in their experiments, we cannot evaluate its potential effect on reducing the number of animals due to underreporting of data. Others [34] reported that in many studies, the number of animals used is not adequately justified, with lack of information about the calculation of sample size (number of animals to be used) being one of the main reasons for the rejection of research protocols by an IACUC in Mexico.[27]

In terms of improvement, 63% of respondents consider that animals can experience negative effects during research use, and they would be willing to use methods that minimize their pain or suffering. However, 32% indicated that they would hesitate to use analgesia or anesthesia if this would affect the experimental results; 60% of respondents would intervene if the procedure had a direct adverse impact on the health of the animals, either by administering a treatment, withdrawing the animal from the experimental group, or performing euthanasia even if these actions incurred a higher cost.[10,58] This is equivalent to using a humane endpoint, which is an indispensable element of improving all experimental procedures with animals by minimizing or mitigating pain or distress and increasing welfare.[1,9] In a previous study carried out in Mexico, the methods used as an endpoint were ethically questionable or were not those recommended in the regulations; this was another principal cause of protocol rejection or non-approval by the IACUC.[3]

The respondents generally showed a reluctance to IACUCs due to the formalities involved in obtaining their opinions and delays in being able to initiate experiments. However, in addition to ensuring the ethical and humane use of animals, the structure and function of IACUCs puts the committees in a unique position to contribute to the quality of the scientific work performed at their institution. The IACUC can be an effective partner in maintaining the quality of results as related to sound experimental design and strategy by providing rigorous and comprehensive evaluation criteria and encouraging responsible research practices.[38]

In terms of IACUC member profiles, some authors report that members of an ethics committee have a limited view of both animal ethics and the proper use of animals in research, suggesting that members should receive more up-to-date information on these topics.[28,29] Given that scientific knowledge is important in the decision-making process when reviewing the appropriateness of research that uses animals, ethical evaluation of the use of animals for biomedical research is a complex process that cannot be reduced to decisions about technical issues such as the type of housing, the environment, surgery protocols, and euthanasia, among others.[28] Others[54] stress that an ethical evaluation requires good reasoning skills, free discussions that can involve disagreements, knowledge of scientific concepts, and an understanding of legal and ethical principles. All these factors should contribute to how committee members evaluate the available information in forming their conclusions.

Although 81% of the respondents are aware of NOM-062-ZOO-1999 "Technical specifications for the production, care and use of laboratory animals",[13] researchers do not appear to have a clear knowledge of its content, despite the fact that the specifications incorporate a variety of standards that affect many areas of animal research, as follows: 1) specific functional areas in both public and private animal facilities; 2) acquisition of animals (housing and nutrition requirements by species); 3) obligations of the institution (operation, personnel, biosafety, transfer of animals, characteristics and care, and 4) guidelines on experimental techniques (anesthesia, analgesia, administration of fluids and other substances, blood extraction and euthanasia).[2]

Mexican regulations do not have a specific requirement for a literature review, and only 2% of the respondents stated that the Guide for the Care and Use of Laboratory Animals informed their experimental or animal care procedures. This is a low percentage considering the information gaps that exist in Mexican regulations, and the fact that The Guide is an internationally known document that undergoes periodic updates and covers a wide scope of research species.[39] In addition, the seventh title of Mexico's Regulations of the General Health Law on Health Research emphasizes that research should be designed in such a way as to avoid animal suffering as much as possible, keeping them in adequate animal facilities based on the species, body conformation, habits, postural preferences and locomotor characteristics of the animals. An exception can be invoked when the experimental variables justify other situations. Moreover, the Regulations stipulate that the animal facility should be supervised by qualified and competent professionals in the field.[8]

In order to promote the use of ethically acceptable methodologies in animal research protocols, an ethics committee must evaluate them. The criteria considered relevant by researchers and IACUC members during the development and evaluation of animal research protocols were animal model selection (92%), scientific validity (89%), the number of animals (89%), the duration of the experiment (85%) and the use of appropriate sampling routes, frequency, and volume (86%). Although these percentages are high, the results also suggest a lack of uniformity in criteria that are used when researchers develop protocols or IACUCs evaluate them.

In essence, ethical evaluation of the use of animals in research involves weighing the harms and benefits, assessing the need, justification for animal use, and methodological processes.[12,44] Directive 2010/63/EU of the European Parliament and of the Council of the European Union explicitly requires the use of methodologies to ethically evaluate a research project involving animals, requiring an institution to have processes that include "assessing whether harm to animals in terms of suffering, pain, and distress is justified by the expected outcomes taking into account ethical considerations and benefiting humans, animals or the environment".[16]

Conclusions

The current study is one of the few existing studies that address the ethical attitudes of the scientific community in Mexico that uses animals for research purposes. Our study provides evidence that despite having some of the most robust animal research legislation in Latin America,[63] some institutions in Mexico still do not have committees to evaluate research proposals; this lack significantly compromises the application of bioethical principles (3Rs) to research. IACUCs are just beginning to consider the implementation of programs that monitor the correct use of animals after protocol approval. Although our findings are largely limited to data from Mexico City, this is the population that dominates biomedical research at the national level, so our results likely reflect the situation in other states of the country. Many areas can be improved, such as the incorporation of methodologies that gradually

contribute to the replacement of animals, mainly in teaching. Likewise, sources of information related to ethics and welfare should be identified to foster implementation of the 3Rs. Several proposed ethical approaches have not yet been implemented. These include the concepts 3S, 3R, 3V, and 6P. The 3Ss are Good Science, Good Sense and Good Sensibilities, the 3Vs emphasize the Validation aspect of animal models, The 4Fs, proposed a framework and several fundamental principles that emphasize the responsibility of the investigator. The 6Ps balances the 2 valued facets of society, social benefit, and animal welfare.[64]

Protocol evaluation requires greater awareness of growing concerns about the poor reproducibility of animal experiments. This lack of reproducibility might be mitigated if guidelines were followed, and necessary considerations were taken into account in the design phase. Adoption of guidelines such as PREPARE that seek to standardize planning criteria are consistent with the promotion of sound and ethical scientific practice, and encourage collaborations between researchers, academia, industry, and organizations in charge of animal welfare. Although a larger sample size could provide additional insights, the current data promotes the use of strategies to continue to improve the current legal framework and promote the development of training programs and guidelines to improve effectiveness of IACUCs.

Acknowledgments

We thank the researchers who participated in this study and Ms. Sara Claudia Herrera-García for her contributions to the development of this work. The authors have no conflicts of Interest with regard to this work.

Funding

Anayantzin Heredia-Antunez (CVU 288530) obtained a scholarship as part of the project financed by the National Council of Science and Technology (Conacyt), "Ethical questions about the use of animals in research in Mexico: alternatives and proposals".

References

1. **3Rs-Centre Utrecht Life Sciences.** 2020. Humane endpoints. 1–7.
2. **de Aluja AS.** 2002. Animales de laboratorio y la norma oficial Mexicana (NOM-062-ZOO-1999). Gac Med Mex **138**:295–298.
3. **Antúnez APH, Cantón BV, Santillán-Doherty P.** 2021. Challenges for ethics committees in animal research. Mexican experience. Rev Bioet Derecho **51**:99–121. https://doi.org/10.1344/rbd2021.51.32563.
4. **Barré-Sinoussi F, Montagutelli X.** 2015. Animal models are essential to biological research: issues and perspectives. Future Sci OA **1**:fso.15.63. https://doi.org/10.4155/fso.15.63.
5. **Baumans V.** 2004. Use of animals in experimental research: An ethical dilemma? Gene Ther **11** S1:S64–S66. https://doi.org/10.1038/sj.gt.3302371.
6. **Brønstad A, Newcomer CE, Decelle T, Everitt JI, Guillen J, Laber K.** 2016. Current concepts of Harm–Benefit Analysis of Animal Experiments – Report from the AALAS–FELASA Working Group on Harm–Benefit Analysis – Part 1. Lab Anim **50 1_suppl:**1–20. https://doi.org/10.1177/0023677216642398.
7. **Browning H, Veit W.** 2020. Improving invertebrate welfare. Anim Sentience **5**. https://doi.org/10.51291/2377-7478.1585.
8. **Camara de diputados del H Congreso de la Unión.** 2014. Reglamento de la Ley General de Salud en Materia de Investigaciones para la Salud. Ley Gen Salud DOF 02-04-:1–31.
9. **Canadian Council on Animal Care.** 2022. CCAC guidelines: Identification of scientific endpoints, humane intervention points, and cumulative endpoints. Ontario.
10. **Carbone L, Austin J.** 2016. Pain and Laboratory Animals: Publication Practices for Better Data Reproducibility and Better Animal Welfare. PLoS One **11**:e0155001. https://doi.org/10.1371/journal.pone.0155001.
11. **CCAC.** Consejo Canadiense de Cuidado Animal: Directrices.
12. **Demers G, Griffin G, Vroey G De, Haywood JR, Zurlo J, Bédard M.** 2006. Harmonization of Animal Care and Use Guidance. Science **312**:700–701. https://doi.org/10.1126/science.1124036
13. **Diario Oficial de la Federacion.** 2001. Norma Oficial Mexicana, NOM-062-ZOO-1999, Especificaciones técnicas para la producción, cuidado y uso de los animales de laboratorio. México.
14. **Direccion General de Salud Animal.** 2022. Directorio de bioterios autorizados.
15. **Doke SK, Dhawale SC.** 2015. Alternatives to animal testing: A review. Saudi Pharm J **23**:223–229. https://doi.org/10.1016/j.jsps.2013.11.002.
16. **European Parliament Council of the European Union.** 2010. Directive 2010/63/EU. Off J Eur Union **53**:33–79.
17. **Ewart L, Apostolou A, Briggs SA, Carman CV, Chaff JT, Heng AR, Jadalannagari S, Janardhanan J, Jang K-J, Joshipura SR, Kadam MM, Kanellias M, Kujala VJ, Kulkarni G, Le CY, Lucchesi C, Manatakis DV, Maniar KK, Quinn ME, Ravan JS, Rizos AC, Sauld JFK, Sliz JD, Tien-Street W, Trinidad DR, Velez J, Wendell M, Irrechukwu O, Mahalingaiah PK, Ingber DE, Scannell JW, Levner D.** 2022. Performance assessment and economic analysis of a human Liver-Chip for predictive toxicology. Commun Med **2**:154. https://doi.org/10.1038/s43856-022-00209-1.
18. **Ferrara F, Hiebl B, Kunzmann P, Hutter F, Afkham F, LaFollette M, Gruber C.** 2022. Culture of care in animal research – Expanding the 3Rs to include people. Lab Anim **56**:511–518. https://doi.org/10.1177/00236772221102238.
19. **Franco NH, Sandøe P, Olsson IAS.** 2018. Researchers' attitudes to the 3Rs—An upturned hierarchy? Puebla I, editor. PLoS One **13**:e0200895. https://doi.org/10.1371/journal.pone.0200895
20. **Gaceta Oficial de la Ciudad de México.** 2002. Ley de proteccion a los animales de la Ciudad De México.
21. **Germán N-H, Rosemarie B.** 2019. A survey in Mexico about ethics dumping in clinical research. BMC Med Ethics **20**:38. https://doi.org/10.1186/s12910-019-0378-6.
22. **Google.** 2023. Google Forms.
23. **Greene ME, Pitts ME, James ML.** 2007. Training Strategies for Institutional Animal Care and Use Committee (IACUC) Members and the Institutional Official (IO). ILAR J **48**:131–142. https://doi.org/10.1093/ilar.48.2.131.
24. **Grimm H, Olsson IAS, Sandøe P.** 2019. Harm–benefit analysis – what is the added value? A review of alternative strategies for weighing harms and benefits as part of the assessment of animal research. Lab Anim **53**:17–27. https://doi.org/10.1177/0023677218783004.
25. **Halley AC.** 2022. The tempo of mammalian embryogenesis: Variation in the pace of brain and body development. Brain Behav Evol **97**:96–107. https://doi.org/10.1159/000523715.
26. **Hansen LA.** 2013. Institution animal care and use committees need greater ethical diversity. J Med Ethics **39**:188–190. https://doi.org/10.1136/medethics-2012-100982.
27. **Heredia Antúnez AP, Cantón BV, Santillán Doherty P.** 2021. Retos de los comités de ética en investigación en animales. Experiencia de México. Rev Bioet Derecho **51**:99–121. https://doi.org/10.1344/rbd2021.51.32563.
28. **Houde L, Dumas C, Leroux T.** 2003. Animal ethical evaluation: An observational study of Canadian IACUCs. Ethics Behav **13**:333–350. https://doi.org/10.1207/S15327019EB1304_2.
29. **Houde L, Dumas C, Leroux T.** 2009. Ethics: Views from IACUC members. Altern Lab Anim **37**:291–296. https://doi.org/10.1177/026119290903700311.
30. **Hvitved AN.** 2019. Engaging ethicists in animal research policy-making. ILAR J **60**:318–323. https://doi.org/10.1093/ilar/ilz023.
31. **INAI.** 2020. Consulta Pública. Inst Nac Transparencia, Acceso a la Inf y Protección Datos Pers.
32. **Johns Hopkins Bloomberg School of Public Health.** Center for Alternatives to Animal Testing.
33. **Khabib MNH, Sivasanku Y, Lee HB, Kumar S, Kue CS.** 2022. Alternative animal models in predictive toxicology. Toxicology **465**:153053. https://doi.org/10.1016/j.tox.2021.153053.

34. **Kilkenny C, Parsons N, Kadyszewski E, Festing MFW, Cuthill IC, Fry D, Hutton J, Altman DG.** 2009. Survey of the quality of experimental design, statistical analysis and reporting of research using animals. PLoS One **4**:e7824. https://doi.org/10.1371/journal.pone.0007824
35. **Klein C, Barron AB.** 2016. Insects have the capacity for subjective experience. Anim Sentience **9**. https://doi.org/10.51291/2377-7478.1113.
36. **Koepsell DR, Ruiz de Chávez MH.** 2015. Etica de la investigacion. Primera. CONBIOETICA, editor. Mexico.
37. **Luis Humberto FC.** 2014. Diez años de apoyo a la Investigación Científica Básica por el CONACYT. Perf Latinoam **22**:55–76.
38. **Mohan S, Huneke R.** 2019. The role of IACUCs in responsible animal research. ILAR J **60**:43–49. https://doi.org/10.1093/ilar/ilz016.
39. **National Research Council.** 2011. Guide for the care and use of laboratory animals. Lab Anim **327**:220–240.
40. **NC3Rs.** 2022. How many animals are used in research?
41. **NC3Rs.** 2023. The 3Rs.
42. **NC3Rs.** 2023. National Centre for the Replacement, Refinement and Reduction of Animals in Research.
43. **NORECOPA.** 2023. Norway's National Consensus Platform.
44. **Plous S, Herzog H.** 2001. Reliability of protocol reviews for animal research. Science **293**:608–609. https://doi.org/10.1126/science.1061621
45. **Robinson NB, Krieger K, Khan FM, Huffman W, Chang M, Naik A, Yongle R, Hameed I, Krieger K, Girardi LN, Guadino M.** 2019. The current state of animal models in research: A review. Int J Surg **72**:9–13. https://doi.org/10.1016/j.ijsu.2019.10.015.
46. **Russel WMS, Burch RL.** 1960. The principles of humane experimental technique. Med J Aust **1**:500–500. https://doi.org/10.5694/j.1326-5377.1960.tb73127.x.
47. **Scalese RJ, Issenberg SB.** 2005. Effective use of simulations for the teaching and acquisition of veterinary professional and clinical skills. J Vet Med Educ **32**:461–467. https://doi.org/10.3138/jvme.32.4.461.
48. **Silverman J, Lidz CW, Clayfield J, Murray A, Simon LJ, Maranda L.** 2017. Factors influencing IACUC decision making: Who leads the discussions? J Empir Res Hum Res Ethics **12**:209–216. https://doi.org/10.1177/1556264617717827.
49. **Smith JA.** 1991. A question of pain in invertebrates. ILAR J **33**:25–31. https://doi.org/10.1093/ilar.33.1-2.25.
50. **Stafleu FR, Tramper R, Vorstenbosch J, Joles JA.** 1999. The ethical acceptability of animal experiments: A proposal for a system to support decision-making. Lab Anim **33**:295–303. https://doi.org/10.1258/002367799780578255.
51. **StataCorp.** 2015. Stata Statistical Software: Release 14. College Station (TX): StataCorp LP.
52. **Tannenbaum J, Bennett BT.** 2015. Russell and Burch's 3Rs then and now: The need for clarity in definition and purpose. J Am Assoc Lab Anim Sci **54**:120–132.
53. **Taylor K, Alvarez LR.** 2019. an estimate of the number of animals used for scientific purposes worldwide in 2015. Altern Lab Anim **47**:196–213. https://doi.org/10.1177/0261192919899853.
54. **Thomson A.** 2000. Critical reasoning in ethics: A practical introduction. Choice Rev Online **37**:37-3857-37–3857. https://doi.org/10.5860/CHOICE.37-3857
55. **USDA APHIS.** 2023. Animal Welfare Act.
56. **van der Valk J, Dewhurst D, Hughes I, Atkinson J, Balcombe J, Braun H, Gabrielson K, Gruber F, Miles J, Nab J, Nardi J, van Wilgenburg H, Zinko U, Zurlo J.** 1999. Alternatives to the use of animals in higher education. Altern Lab Anim **27**:39–52. https://doi.org/10.1177/026119299902700105.
57. **Veening-Griffioen DH, Ferreira GS, Boon WPC, Gispen-de Wied CC, Schellekens H, Moores EHM, Van Meer PJK.** 2021. Tradition, not science, is the basis of animal model selection in translational and applied research. ALTEX **38**:49–62. https://doi.org/10.14573/altex.2003301.
58. **Williams WO, Baneux P.** 2022. Humane Intervention Points: Refining endpoint terminology to incorporate non-euthanasia intervention options to improve animal welfare and preserve experimental outcomes. Lab Anim **56**:482–489. https://doi.org/10.1177/00236772221090801.
59. **Würbel H.** 2017. More than 3Rs: The importance of scientific validity for harm-benefit analysis of animal research. Lab Anim (NY) **46**:164–166. https://doi.org/10.1038/laban.1220.
60. **Zemanova MA, Knight A, Lybæk S.** 2021. Educational use of animals in Europe indicates reluctance to implement alternatives. ALTEX **38**:490–506. https://doi.org/10.14573/altex.2011111.
61. **Speaking of Research.** 2023.
62. **Abbott A.** 2010. Basel Declaration defends animal research. Nature **468**:742. https://doi.org/10.1038/468742a.
63. **Petkov CI, Flecknell P, Murphy K, Basso MA, Mitchell AS, Hartig R, Thompson-Iritani S.** 2022. Unified ethical principles and an animal research "Helsinki" declaration as foundations for international collaboration. Curr Res Neurobiol **3**:100060. https://doi.org/10.1016/j.crneur.2022.100060
64. **Smith AJ, Clutton RE, Lilley E, Hansen KEA, Brattelid T.** 2018. PREPARE: Guidelines for planning animal research and testing. Lab Anim **52**:135-141. https://doi.org/10.1177/0023677217724823.
