# Supplementary material for: Laboratory animal ethics education improves medical students' awareness of laboratory animal ethics
Source: BMC Med Educ. 2024 Jul 1;24:709. doi: 10.1186/s12909-024-05703-9 (PMC11218205; doi:10.1186/s12909-024-05703-9)
Supplement: Supplementary file 1 — Supplementary Material 1. [file 12909_2024_5703_MOESM1_ESM.zip › The revised table/Knowledge, Attitudes, and Practices on Ethics in Biomedical Animal Research in Mexico_1_9_translate_20240527164450.pdf]

美国实验动物科学协会的杂志
版权所有2023
由美国实验动物科学协会提供

第62卷，
2023年11月6日
，第478-486页

道德规范方面的知识、态度和实践在墨西哥的生物医学动物研究中

埃雷迪亚-安图涅斯，[1,#]米格尔・加拉德-洛佩兹[2,#]伊丽莎白・特莱斯-巴列斯特罗斯，[3]还有比阿特丽斯・范达-坎顿[3,*]

在生物医学研究中使用的减轻对动物的伤害的最被广泛接受的伦理概念被称为3Rs，它指的是替代、减少和改进。我们研究的目的是确定墨西哥研究人员在制定动物研究方案时考虑的伦理和监管标准，以及伦理委员会成员在评估和批准这些方案时使用的标准。我们向来自不同研究机构的300名个人分发了一份调查，并收到了179名研究人员和伦理委员会成员的回答，关于他们在基于3r的研究中使用动物的知识、态度和实践的问题。所获得的答复表明，受访者知道3R的概念，并且他们声称应用了这些原则。然而，反应显示反对使用研究、测试和教学（66%）。19%的研究人员报告说，他们的机构没有一个综合的机构动物护理和使用委员会（IACUC）。约80%的受访者知道墨西哥的法规。研究人员和IACUC成员对3Rs的知识和应用是动物研究中的一个基本概念。这些知识有助于使用与在研究中使用动物相关的伦理标准、态度和实践。

缩写和首字母缩写：机构动物护理和使用委员会（IACUC）

DOI： 10.30802/AALAS-JAALAS-23-000012

介绍

动物研究对于理解生物过程和疾病机制、开发和测试疫苗以及寻找更多更好的有利于人类和其他动物的治疗方法至关重要。[5,45]根据欧盟的数据，2019年有超过1160万只所有类型的动物被用于研究目的，而在美国，同年大约有80万只动物被用于研究目的。由于汇编所使用的动物数量的不同，这些数据不具有可比性，但它们仍然反映了用于这一目的的动物数量。[61]然而，这些数据被低估了，因为它们不包括老鼠、大鼠、鱼或鸟类。[40,55]2019年，超过三分之二的动物被用于基础研究（45%）和应用研究（23%），而约四分之一（23%）的动物被用于药物和其他化学测试，以满足监管要求。其他形式的动物使用包括生物制剂的常规生产，如疫苗和教学。[53,61]

一般来说，获得世界范围内研究中使用的动物数量的准确计数是困难的，因为一些国家没有跟踪使用的动物的数量。[53]虽然动物仍然是生物医学研究的进展是必要的，但动物研究必须在一个伦理框架内进行。

提交：2023年2月10日。修订要求：2023年3月23日。接受：2023年6月6日。

[1]墨西哥国立自治大学医学、牙科和健康科学研究生，墨西哥城，墨西哥；[2]墨西哥莫雷洛斯国家公共卫生研究所传染病研究中心，墨西哥莫雷洛斯；和[3]墨西哥国立自治大学兽医和动物技术学院，墨西哥城

**通讯作者。电子邮件 daktari@unam.mx*

#这些作者对这项工作的贡献相等。共同第一作者的顺序是由两位共同第一作者之间的讨论和相互一致来确定的。

3Rs的概念已成为动物研究的伦理基准，并已在几乎所有国家的立法中明确或含蓄地采用。[46]3Rs最初是为了减少研究程序对动物的危害和负面影响。

替代包括用替代品取代动物，包括动物组织或伦理获得的人体组织、计算机模型、芯片上的所谓器官，以及硅技术，如计算机建模。细胞培养、微生物和无脊椎动物也可以被使用。在实践中，替换是3Rs中最难实现的。

减少是使用最少的符合统计可靠研究的动物，并避免不必要的重复实验。

改进是使用侵入性较小的技术和程序，有效地使用麻醉和镇痛来减少疼痛、焦虑和不适，以及环境丰富的项目，以改善动物从出生到死亡的福利。[40,41,52]

墨西哥有大约60个动物设施，它们是由农业部（SADER）的卫生、安全和农业食品质量服务处（参议院）注册和授权的。这些设施中的动物，如啮齿动物、兔子、鸟类、鱼、爬行动物、猪、反刍动物、马和非人类灵长类动物等。其中约60%隶属于高等教育机构（HEI），35%隶属于私营企业，5%隶属于国家卫生机构（卫生部、墨西哥社会保障研究所等）。[14]，墨西哥官方标准，NOM-062-ZOO-1999年，“实验动物的生产、护理和使用的技术规范”建立

允许使用它们所必须满足的条件。根据规定，动物只能用于研究只有在1)研究是必要的和知识不能通过其他手段，2)替代动物没有（例如，体外培养、模拟器、数学模型），和3)结果不能预测和类似的研究没有发表。[13,16,39]

在一些国家，动物实验必须得到一个伦理委员会的授权，该委员会有正式任命的成员，他们从伦理和方法的角度评估研究方案。[29]在墨西哥，在研究开始之前必须得到IACUC的批准。如果研究者的机构没有IACUC，他/她可以请求其他确实有IACUC的机构的支持，以获得此批准。[13]在研究中使用动物的伦理评估包括权衡对动物的危害与预期的好处、使用动物的理由、实验的重要性和所采用的方法。[44]在墨西哥，有害效益分析通常不包括在动物研究方案中，因为规定中没有要求。因此，重点是是否符合3Rs。相比之下，欧洲指令要求动物研究方案从利于弊的角度进行评估。S.实验动物的护理和使用指南要求iacuc进行类似的评估。[16]动物实验监管的基本伦理原则是，对动物或人可能得到的好处应该超过对实验动物造成的伤害和痛苦；因此，研究人员必须明确阐明要进行的研究的目的，并解释为什么它是重要的。[48,50]本研究的目的是确定研究人员和伦理委员会成员在墨西哥制定和评估动物研究方案时所考虑的伦理和监管标准。

材料和方法

设计和研究人群。这项横断面描述性研究是在2018年至2021年期间针对墨西哥大学、卫生部机构和机构药物检测实验室的动物护理和使用委员会（IACUCs）成员的研究人员和调查进行的。

这项调查包括了研究人员和伦理委员会的成员，并通过亲自和在线的方式进行(使用谷歌表单平台[22]).由于墨西哥采用的SARS-COV-2社交距离政策，这种虚拟方法是必要的。参与者是通过使用国家透明平台和国家动物设施目录从公共和私人研究机构（大学、卫生部机构和制药公司）中挑选出来的（图1）。[14,31]国家透明平台（PNT）一般允许公民向墨西哥三级政府的各个受监管实体提交获取公共信息和访问、纠正、取消、反对或移植个人数据（ARCOP）的请求。

调查包含28项，分为6部分： 1)研究人员和/或委员会成员，2)机会在IACUC，3)选择动物模型用于研究，4)动物研究的伦理原则（3Rs），5)应用的监管框架的知识，和6)标准批准或开发一个研究协议。这些问题已经得到了验证[19,21]并为我们的研究进行了修改和试点。

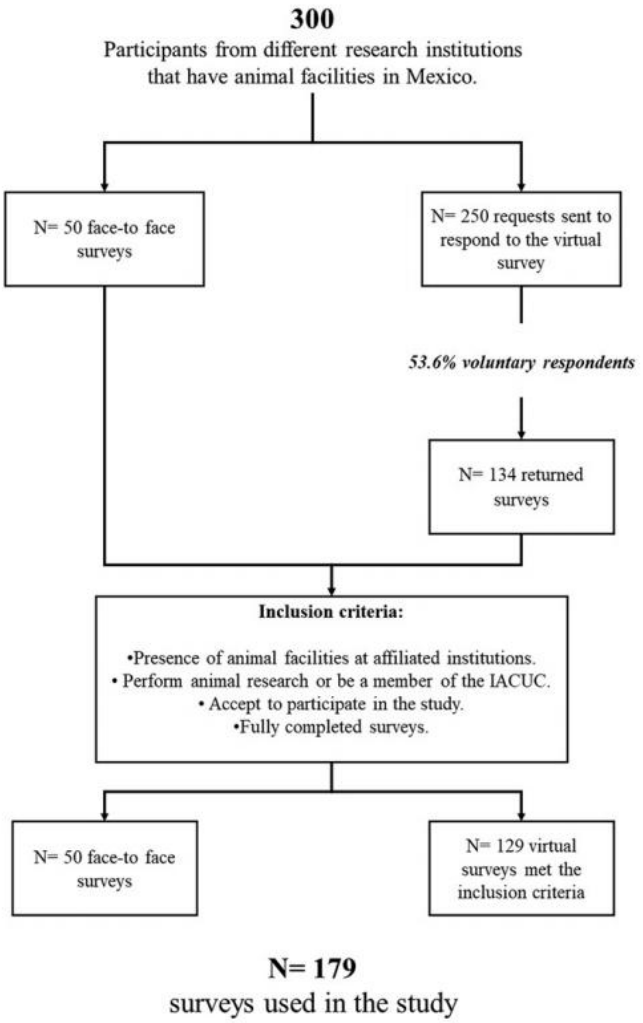

图1。调查方法总结流程图。

数据分析。我们分析了感兴趣的变量，以获得集中趋势、频率和比例的测量值。只提供了描述性的统计数据。数据分析采用数据科学的统计程序（STATA v.15，Stata Corp，大学站，TX）。[51]

伦理批准声明。该研究得到了墨西哥医学、牙科和健康科学硕士和博士项目的伦理和研究委员会的批准。不2019年5月。对答复者及其所属机构保持保密性。

结果

我们进行了50次面对面调查，对250项邮件请求的回复率为54%，结果得到134项返回调查，其中129项符合纳入标准。所有被调查的人都是墨西哥城地铁的研究人员。

在179项可用的调查中（50项面对面调查和129项虚拟调查）。49%来自女性，51%来自男性；平均年龄为40岁（sd，13岁），年龄从21岁到90岁。附属机构如下： 51%在大学工作，27%在医院和卫生部机构工作，4%在制药行业工作，4%在私营行业工作，13%没有说明他们所属的部门。在受访者中，88%的人与动物一起工作，12%的人以前有过与动物打交道的经验。

81%的人报告说他们的机构患有IACUC。在剩下的19%（34人）的参与者中，有39%（13人）报告说

IACUC的创建过程正在进行中，36%（12）不知道为什么IACUC不存在，24%给出了IACUC不存在的原因（缺乏机构（15%，5），或教师（6%，2）利息，以及其他原因（3%，1））。 只有15%的受访者是IACUC的成员。在受访者中，18%接受过一些生物伦理学方面的培训（课程、证书和研究生学位等）（表1）。**用动物来进行的研究的类型。**研究人员被要求指出他们曾经使用动物和没有使用动物的研究领域。动物使用最常见的领域包括基础研究（60%）、为人类利益的应用研究（45%）、以及在教育和培训方面（42%）。受访者从未使用过动物的最常见领域包括毒理学研究（78%）、药物和/或疫苗的开发、生产、控制或测试（74%）、疾病诊断（62%）和为动物的利益而进行的应用研究（53%）（表2）。由于个人对同一问题的多次回答，报告的百分比总数超过100%。

替代使用传统的研究物种。受访者被要求指出哪些可能的替代典型的研究物种是最可行的。这些排名如下：体外模型，67%；使用无脊椎动物，49%；使用动物胚胎，38%；虚拟模拟或模型，37%；农场动物，35%；芯片上的生物芯片或器官，32%，尸体，17%）。

对研究中使用的动物物种的伦理态度。研究人员被要求对他们对使用各种动物进行研究的接受程度进行评分。“完全可接受”或“可接受”的选择是动物胚胎（100%）、猪（97%）、斑马鱼（89%）、果蝇（84%）和大鼠（80%）。

最不可接受（“完全不可接受”或“不可接受”）的是黑猩猩（100%）、狗（51%）和猪（2%）。完整的数据见表3。

在179名受访者中，接受或不接受在研究中使用某些动物物种的原因如下：法律限制，59%；对疼痛的敏感程度，32%；系统发育接近人类，27%；人-动物与该物种的联系强度为6%。

3Rs的应用。关于研究动物的替换，66%的受访者同意，由于缺乏获得同样可靠的结果的信心，因此无法实施脊椎动物模型的替换。此外，20%的人认为关于替代方法的信息并不容易找到。然而，73%的人同意，如果研究方案的目标可以通过使用替代方法来实现，他们将应用这些方法。关于研究动物数量的减少，94%的人同意进行广泛的文献综述，以证明他们的项目的合理性，并避免不必要的重复，87%的人声称对他们的实验中使用的动物数量使用了统计理由。

针对细化，83%的人同意使用麻醉和镇痛在对动物造成不适和疼痛的侵入性实验中是必不可少的。然而，如果它们的使用可能会干扰结果，19%的人会犹豫是否使用它们。此外，75%的人同意，当一个

表1。研究人群的特征

变量	n = 179
年龄(y)（平均值、标准差、范围）	40, 13, (21 – 90)
性别	
女性的	87 (49)
男	92 (51)
受访者	
在研究中使用动物。	157 (88%)
不要在研究中使用动物	22 (12%)
IACUC委员会成员	
是	26 (15%)
不	153 (86%)
参加过生物伦理学课程	
是	32 (18%)
不	147 (82%)
机构有一个IACUC	
是	145 (81)
不	34 (19)
附属机构	
学术界	92 (51%)
制药行业	7 (4%)
医院	49 (27%)
私人公司	7 (4%)
在机构没有IACUC（n=34）	
缺乏机构利益	5 (15%)
研究人员对创建IACUC的过程缺乏兴趣，	2 (6%)
并不知道原因	13 (39%)
另一个原因	12 (36%)
	1 (3%)

表2。为上述目的进行动物实验的频率

目的：n = 179	从不	几乎从来没有	有时	几乎总是	总是	平均态度值	95% CI
开发、生产、控制或测试药物和/或疫苗	74%	12%	6%	5%	3%	1.5	1.3-1.6
毒理学研究	78%	13%	7%	0.5%	1.7%	1.3	1.2-1.4
疾病诊断	62%	11%	22%	2%	3%	1.7	1.5-1.8
教育和培训	32%	8%	16%	13%	31%	3.0	2.7-3.2
基础研究	13%	2%	5%	20%	60%	4.1	3.9-4.3
应用研究，造福于人类	27%	3%	6%	19%	45%	3.5	3.2-3.7
应用于动物效益的研究	52%	24%	14%	2%	8%	1.9	1.7-2.0

受访者如果回答“Never”，就会得到1分；2)“几乎从不”；3)“有时”；4)“几乎总是”；5)“总是”。因此，每个语句的平均分接近5分表示频繁使用动物物种，而接近1分表示不再使用。95% CI，95%置信区间。

表3。在基于物种的实验中接受或拒绝使用动物的程度

物种	完全不可接受	不可接受	无意见	可接受	完全可以接受	平均态度值	95% CI
胚胎（不同动物种类）	0%	0%	0%	0%	100%	5.0	. – .
公狗	21%	31%	15%	22%	11%	2.7	2.5-2.9
老鼠	0%	0%	20%	39%	41%	4.2	4.1-4.3
果蝇（*果蝇*）	0%	0%	12%	14%	74%	4.6	4.5-4.7
猪	1%	1%	0%	37%	61%	4.5	4.4-4.6
斑马鱼	0%	0%	11%	37%	52%	4.4	4.3-4.5
黑猩猩	19%	81%	0%	0%	0%	1.8	1.7-1.9

“完全不可接受”的回答分为1分；2）“不可接受”；3）“无意见”；4）“可接受”；5）“完全可接受”。因此，每个陈述的平均分接近5分，就表示他们对动物物种的态度很积极 得分接近1表示态度消极。95% CI，95%置信区间。

实验程序对动物的健康和福利有直接的负面影响，因此应该终止实验程序，对动物实施安乐死，从研究中删除，或给予治疗，即使实验还没有结束。在选择安乐死方法的情况下，25%的人认为选择该方法不应该考虑成本（表4）。

IACUC的性能。当被问及IACUC的权威时，69%的人同意IACUC应该有权在动物健康和/或福利时停止实验会妥协。此外，62%的人同意遵守IACUC的建议应由研究人员自行决定。关于IACUC的构成，85%的人认为IACUC应该只包括该领域的医生或研究人员，87%的人认为IACUC的成员应该接受生物伦理学主题方面的额外培训。

关于IACUC的使用，85%的人认为在制定研究方案时应咨询委员会成员，以获得相关意见。最后，73%的人强烈同意委员会应该批准一名调查人员

表4。关于使用动物进行生物医学研究

你在多大程度上同意以下陈述？(n = 179)	强烈反对	不同意	不意见	同意	非常同意	平均态度值	95% CI
我不愿意应用我的方法的任何改变来替代脊椎动物，因为我还没有信心获得与其他模型同样可靠的结果	1%	7%	26%	37%	29%	3.8	3.7-3.9
在我的研究线中，关于动物使用的替代方法的信息很容易找到和获得，	12%	21%	47%	17%	3%	2.7	2.6-2.9
在导致动物疼痛或不适的侵入性实验中，在手术过程中和术后使用麻醉和镇痛是必不可少的	1%	0%	16%	20%	63%	4.4	4.3-4.5
止痛药应该在产生动物疼痛的程序中使用，即使它们改变了动物病情的可能结果	24%	26%	32%	12%	6%	3.4	3.3-3.6
当进行实验程序时，动物出现明显的疼痛和健康状况恶化，应从组中移除，给予治疗，或安乐死，即使实验时间没有过去	1%	3%	21%	16%	59%	4.2	4.1-4.4
如果要采用动物死亡的方法，则应选择最经济的方法	25%	24%	26%	9%	16%	2.6	2.4-2.8
如果这些研究目标可以通过尸体、孤立的器官、细胞培养物、模拟器或自然动物模型来实现，那么它们就应该立即得到实施	6%	5%	16%	14%	59%	4.14	3.9-4.3
在启动或评估一个项目之前，应该对科学文献进行彻底的审查，以获得原始知识	0%	0%	5%	13%	82%	4.75	4.6-4.8
为了确定实验组中的动物数量，应该进行包括实验组的数量和待比较变量在内的统计检验	1%	3%	8%	11%	77%	4.58	4.4-4.7

回答“强烈不同意”的受访者得到1分；2)“不同意”；3)“没有意见”；4)“同意”；5)“强烈同意”。因此，每个陈述的平均分接近5分表示对动物研究的积极态度，而接近1分表示消极态度。95% CI，95%置信区间。

表5。关于IACUC功能的意见

你在多大程度上同意以下陈述？(n = 179)	强烈反对	不同意	无意见	同意	非常同意	平均态度值	95% CI
IACUC必须停止实验当动物福利妥协在进行研究的决定	2%	4%	24%	29%	41%	4.0	3.8-4.1
实施建议IACUC应该留下的自由裁量权研究者	4%	11%	23%	32%	30%	3.7	3.5-3.9
IACUCs不需要评估协议涉及动物的使用的理想的形象	51%	33%	10%	2%	4%	4.2	4.0-4.4
IACUC成员参与协议使用动物的审查应该包括医生或研究人员领域	3%	1%	12%	30%	55%	4.3	4.2-4.4
IACUC成员参与开发或审查协议涉及使用动物必须接受伦理或生物伦理学培训	2%	1%	9%	38%	50%	4.3	4.1-4.4
进行适当的制裁不遵循IACUC建议的研究人员应该暂停实验	0%	10%	18%	42%	30%	3.9	3.7-4.0
为了获得及时和相关的意见，在制定研究方案时应咨询IACUC的一名成员	0%	0%	15%	37%	48%	4.3	4.2-4.4

回答"强烈不同意"的受访者得到1分；2)"不同意"；3)"没有意见"；4)"同意"；5)"强烈同意"。因此，每个陈述的平均分接近5分表示对动物研究的积极态度，而接近1分表示消极态度。95% CI，95%置信区间。

如果实验不符合IACUC发布的要求，则停止实验（表5）。

规定。关于指导方针、文件或法规咨询开发或评估研究协议涉及的使用动物，81%的受访者提到NOM-062-ZOO-1999开放式响应（虽然不是名字），17%不知道参考文档，和2%的人提到美国实验动物的护理和使用指南。[12]

在制定和批准涉及动物的研究方案时所考虑的主要方面。受访者被要求对动物方案中17个元素的重要性进行评级（表X）。最重要的最不重要的，项目排名从最高（92%）到最低（21%）如下：动物模型的选择，科学有效性、使用的动物的数量，实验的持续时间，路线、频率和采样、环境富集程序的使用，入侵程度，人类风险、麻醉药和镇痛药的使用，安乐死的方法（图2）。

讨论

在回答调查的总数（n = 179）中，近50%的受访者在高等教育机构工作，27%在医院工作，较少部分在私立机构工作。这种分布可能与受访者来自墨西哥城有关。根据2020年国家动物设施目录，在国家一级，40%的人在城市，卫生部门中数量最多的大学和机构也在城市。[14]因为我们想对受访者保密，所以我们没有确定受访者是否来自同一机构，包括我们所在的机构，或者来自同一机构的受访者是否有类似的回答。

只有18%的受访者参加过生物伦理学课程，尽管87%的人在研究中使用动物，这表明在研究中，生物伦理学和动物福利方面的培训水平较低。参与开发或评估使用动物的实验方案的个人应考虑他们的管理和护理，以及法律考虑和生物伦理原则。[18,30]这一知识对审查动物研究方案的委员会的成员来说很重要，而且应该包括对有害-利益和伦理问题的评估。[6,24,26]

大约20%的机构使用动物研究、实验，和/或教学没有IACUC尽管墨西哥官方标准NOM-062-ZOO-1999"技术规范生产、护理和使用实验动物"，表明所有机构进行科学研究、技术开发、实验和教学必须形成一个IACUC。[13]此外，《墨西哥城动物保护法》第47条规定，动物实验应按照墨西哥官方标准进行，并在机构生物伦理委员会面前充分证明是合理的。[20]然而，墨西哥现有的墨西哥监管文件都没有要求有动物设施的机构或在研究中使用动物注册IACUC的机构，因此墨西哥没有标准的IACUC指南。监管文件没有讨论IACUC的角色、功能或报告要求，也不包括对所使用动物的数量和类型的报告要求。因此，在墨西哥，在研究中使用动物的报道被低估了。[27]

尽管这是《巴塞尔宣言》的基本原则[62]认识到基础研究不能与应用研究分离，受访者对动物的主要用途80%用于基础研究，64%用于人类应用研究，11%用于动物应用研究。这一结果与墨西哥为

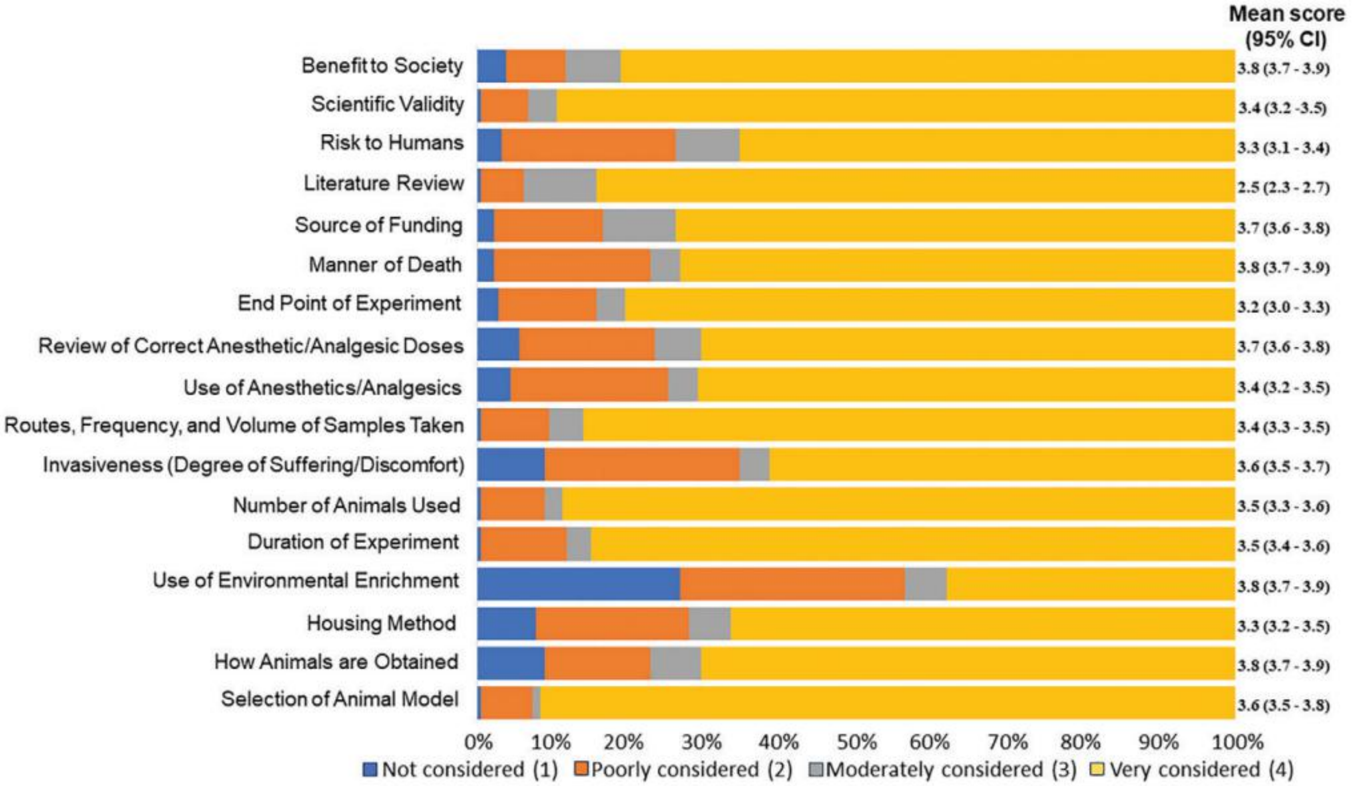

图2。在使用动物的研究方案的开发和评价中存在的因素。每个陈述的平均得分接近4表示在动物研究方案的制定和评估中考虑到的一个因素，而得分接近1表示没有考虑到的一个因素。CI 95：置信区间为95%。

基础研究项目，这可能会也可能不会达到应用知识达到顶峰。37考虑到使用动物模型的理由之一是跨物种相似性，应该鼓励更多有利于非人类动物的研究。4,59我们的研究没有评估对在研究中使用和不使用动物的个体的基于动物的研究的认知差异。

关于使用动物教学协助收购专业能力在生物医学领域，尚不清楚为什么这么多动物仍然使用，尽管多种替代方法已经开发出取代和减少动物的数量为了应用3Rs的原则在教学。47,56一组60发现了一个强烈的信念，一个生物必须用于最佳学习，没有其他模型充分模拟这种经验。更好地对教师进行教育和培训是必要的，以促进更多地使用非动物的替代品，以实现学习目标。

关于在动物研究中使用的替代方法，94%的受访者知道替代方法，其中细胞培养的报道最多。受访者通常对列出的其他替代方案没有信心，并声称关于替代方案的信息很难找到。11,32,42,43尽管有些替代方案存在经济或技术上的限制，17各机构似乎对促进产生动物使用的替代品以满足它们的需求没有什么兴趣。研究人员应该通过多学科的工作，对应用或开发替代方案表现出更大的兴趣。

基于目前研究中使用动物提出的伦理问题，研究结果显示，人们更倾向于使用动物胚胎模型和无脊椎动物模型，尽管这是如何我们获得了胚胎，但不考虑相关的物种。由于胚胎的神经发育不完全和痛觉，这一结果并不意外。25而对苍蝇等无脊椎动物的痛觉和感觉机制也普遍缺乏关注。7,35,49历史上，法律、伦理准则和准则都建议使用无脊椎动物作为脊椎动物的部分替代品。15,33,39另一方面，我们的受访者不太关心猪、斑马鱼和老鼠等脊椎动物；首选物种在管理、饲养和在研究中的广泛使用方面有明显的优势。其他57已经注意到，动物模型的选择很大程度上取决于研究人员基于其个人经验或物种在其机构的可用性的偏好，而不是对他们的研究问题的最合适模型的仔细查询。在制定研究方案时，狗与动物物种的人类关系是受访者最不重要的考虑因素，但狗是最被认为完全不能接受研究的物种。这种看法可能受到了《墨西哥城动物保护法》所规定的法律限制的影响。49)，它禁止将狗和猫用于研究和教学目的。20

关于知识的实现和应用3Rs动物研究协议，66%的受访者怀疑替代方法会帮助他们获得数据一样可靠的通过使用动物，但如果他们确信他们可以实现他们的目标通过使用替代方法，他们会使用它们。也许，实施机构的培训方案和研讨会，以寻找替代方法23是否有助于让更多的人认识到替代方案的有用性

动物研究的方法。很大比例的受访者认为文献综述是研究者伦理承诺的一部分，以避免不必要的重复（94%）。[36]

尽管87%的受访者报告曾进行了统计测试，以证明他们实验中使用的动物数量，但由于数据报告不足，我们无法评估其对减少动物数量的潜在影响。其他[34]报告说，在许多研究中，使用的动物数量没有充分的理由，缺乏关于计算样本量（要使用的动物数量）的信息是墨西哥IACUC拒绝研究方案的主要原因之一。[27]

在改善方面，63%的受访者认为动物在研究使用过程中可能会经历负面影响，他们愿意使用将疼痛或痛苦降到最低的方法。然而，32%的人表示，如果这会影响实验结果，他们会犹豫使用镇痛或麻醉；如果手术对动物的健康有直接不利影响，60%的受访者会进行干预，要么进行治疗，将动物从实验组中退出，要么即使这些行动成本更高也实施安乐死。[10,58]这相当于使用人道终点，这是通过最小化或减轻疼痛或痛苦或增加福利来改善动物所有实验程序的不可或缺的元素。[1,9]在之前在墨西哥进行的一项研究中，作为终点使用的方法在伦理上存在问题，或者不是法规中推荐的方法；这是IACUC拒绝或不批准方案的另一个主要原因。[3]

受访者通常不愿接受iacuc，因为他们的意见，而且无法启动实验。然而，除了确保以伦理和人道的方式使用动物外，iacuc的结构和功能使各委员会处于独特的地位，可以为其机构所进行的科学工作的质量作出贡献。通过提供严格和全面的评估标准和鼓励负责任的研究实践，IACUC可以成为保持与健全的实验设计和策略相关的结果质量的有效合作伙伴。[38]

关于IACUC的成员的简介，一些作者报告说，伦理委员会的成员对动物伦理和在研究中正确使用动物的看法有限，这建议成员应该获得更多关于这些主题的最新信息。[28,29]鉴于科学知识是重要的决策过程时，研究使用动物，伦理评估使用动物进行生物医学研究是一个复杂的过程，不能减少决策技术问题如住房的类型，环境、手术协议，安乐死，等等。[28]其他[54]强调伦理评估需要良好的推理能力，可能涉及分歧的自由讨论，对科学概念的知识，以及对法律和伦理原则的理解。所有这些因素都有助于委员会成员如何评价现有信息以形成结论。

尽管81%的受访者知道NOM-062ZOO-1999年的“实验动物的生产、护理和使用的技术规范”，[13]研究人员似乎对其内容没有明确的了解，尽管该规范包含了影响动物研究的许多领域的各种标准，如下： 1)公共和私人动物设施的特定功能领域；2)动物获取（按物种划分的住房和营养要求）；3)机构的义务（操作、人员、生物安全、动物转移、特性和护理，以及4）实验技术指南（麻醉、镇痛、输液和其他物质、血液提取和安乐死）。[2]

墨西哥的法规对文献综述没有具体的要求，只有2%的受访者表示，实验室动物护理和使用指南告知了他们的实验或动物护理程序。考虑到墨西哥法规中存在的信息差距，以及该指南是一份国际知名的文件，经过定期更新，涵盖了广泛的研究物种，这一百分比很低。[39]此外，第七个标题墨西哥的一般卫生法律卫生研究强调研究应该设计的方式尽可能避免动物痛苦，让他们在足够的动物设施基于物种，身体构象、习惯、姿势偏好和动物的运动特征。当实验变量证明其他情况时，可以调用一个例外。此外，条例规定动物设施应由合格和合格的专业人员进行监督。[8]

为了促进在动物研究方案中使用伦理上可接受的方法，伦理委员会必须对其进行评估。研究人员和IACUC成员在制定和评估动物研究方案时认为相关的标准包括动物模型选择（92%）、科学效度（89%）、动物数量（89%）、实验持续时间（85%）和使用适当的采样路线、频率和体积（86%）。尽管这些比例很高，但结果也表明，当研究人员开发协议或评估机构评估它们时，所使用的标准缺乏一致性。

本质上，是在研究中使用动物的伦理评价 包括权衡弊与利，评估需求， 动物使用的理由，和方法过程。[12,44]指令2010/63/欧盟的欧洲议会和欧盟理事会明确要求使用方法伦理评估涉及动物的研究项目，要求一个机构有流程，包括“评估是否伤害动物的痛苦、痛苦和痛苦是合理的预期结果考虑伦理考虑和造福人类、动物或环境”。[16]

结论

目前的研究是现有的为数不多的、涉及墨西哥科学界使用动物作为研究目的的伦理态度的研究之一。我们的研究提供了证据，尽管在拉丁美洲有一些最有力的动物研究立法，[63]墨西哥的一些机构仍然没有委员会来评估研究提案；这种缺乏严重影响了生物伦理原则（3Rs）在研究中的应用。iacuc刚刚开始考虑在方案批准后监测动物正确使用的项目。虽然我们的发现在很大程度上局限于来自墨西哥城的数据，但这是在国家层面上主导生物医学研究的人口，所以我们的研究结果很可能反映了该国其他州的情况。许多领域可以改进，例如逐步纳入方法

有助于替代动物，主要是在教学方面。同样，应确定与道德和福利有关的信息来源，以促进3r的实施。一些拟议的伦理方法尚未得到实施。这些概念包括概念3S、3R、3V和6P。3Ss是良好的科学，良好的理智和良好的敏感性，3Vs强调动物模型的验证方面，4Fs，提出了一个框架和几个基本原则，强调研究者的责任。6Ps平衡了社会、社会福利和动物福利的两个重要方面。64

方案评估需要更多地认识到对动物实验可重复性差的日益关注。如果遵循指导方针，并在设计阶段考虑到必要的考虑，这种再现性的缺乏可能会得到缓解。采用诸如寻求标准化规划标准的准则与促进健全和伦理的科学实践是一致的，并鼓励研究人员、学术界、工业界和负责动物福利的组织之间的合作。虽然更大的样本量可以提供更多的见解，但目前的数据促进了使用战略，以继续改进目前的法律框架，并促进制定培训方案和准则，以提高iacuc的有效性。

确认信息

我们感谢参与这项研究的研究人员和女士女士。萨拉克劳迪娅埃雷拉-加西亚，感谢她对这项工作的发展的贡献。作者在这项工作方面没有利益冲突。

资金

作为美国国家科学技术委员会(安图尼茨（CVU 288530）资助的项目“关于在墨西哥研究中使用动物的伦理问题：替代方案和建议”的一部分。

参考文献

1. **乌得勒支生命科学中心。**2020.人类的端点。1 – 7.
2. **德阿鲁贾。**2002.墨西哥实验室动物（1062-ZOO-1999）。Gac Med Mex 138:295 – 298.
3. **安图尼兹，广州，桑蒂兰多尔蒂。**2021.动物研究中的伦理委员会面临的挑战。墨西哥的经验。RevBioetDerecho51：99-121。https://doi.org/10.1344/ rbd2021.51.32563.
4. **蒙塔古泰利X。**2015.动物模型对生物学研究至关重要：问题和观点。未来Sci OA 1：fso5.63。.1 https://doi.org/10.4155/fso..15.63
5. **鲍曼斯诉。**2004.在实验研究中使用动物：一个伦理困境？基因Ther11S1：S64-S66。https://doi .org/10.1038/sj.gt.3302371.
6. **Brønstad A，新人CE，皇帝，埃弗里特吉，吉伦，拉伯K.**2016.目前的伤害概念-动物实验的利益分析-来自AALAS-FELASA伤害工作组的报告-利益分析-第1部分。**实验室Anim 50 1_suppl：1-20**。https://doi.org/10.1177/0023677216642398.
7. **勃朗宁，韦特。**2020.改善无脊椎动物的福利。Anim情感5。https://doi.org/10.51291/2377-7478.1585.
8. **这是一个联盟的大会。**2014.负责调查的将军。Ley将军Salud DOF 02-04-：1-31。
9. **加拿大动物保护委员会。**2022.CCAC指南：确定科学终点、人道干预点和累积终点。安大略省
10. **Carbone L，奥斯汀J。**2016.疼痛和实验动物：更好的数据重现性和更好的动物的出版实践 幸福PLoS11：e0155001。https://doi.org/10.1371/journal.pone.0155001.
11. **中国民用航空总局**加拿大动物：指南。
12. **德默斯G，格里芬G，弗罗伊G德，小海伍德，祖洛J，贝达德M.**2006.动物护理和使用指导的协调。科学312：700-701。https://doi.org/10.1126/science.1124036
13. **《联邦政府的官方日报。**2001.《墨西哥官方动画》，11月062年，1999年，作品动画，实验室动画。墨西哥
14. **动物主任主任。**2022.自动生物技术指令。
15. **Doke SK，达瓦尔SC。**2015.动物测试的替代品：一个回顾。沙特医药J23：223-229。https://doi.org/10.1016/ j.jsps.2013.11.002.
16. **欧盟的欧洲议会理事会。**2010.指令2010/63/欧盟。离开欧盟联盟53点33分-79分。
17. **伊瓦特、阿普斯托拉、布里格斯、卡曼、卡夫斯、卡夫斯、卡达斯、卡尼斯、卡尼斯、卡尼斯、斯、斯尼斯、斯卡斯、斯坎纳尔JW、莱夫纳D。**2022.人类肝脏-毒理学预测芯片的性能评估和经济分析。Commun地中海2：154。https://doi.org/10.1038/s43856-022-00209-1.
18. **费拉拉、希布尔、昆兹曼、哈特、阿夫卡姆、拉福莱特、格鲁伯C。**2022.动物研究中的护理文化-扩大3Rs到包括人。实验室Anim56：511-518。https://doi.org/10.1177/00236772221102238.
19. **弗朗哥NH，Sandøe P，Olsson IAS。**2018.研究人员对3r的态度-一个颠覆的等级制度？Puebla I编辑。PLoS113：e0200895。https://doi.org/10.1371/journal.pone895.0200
20. **墨西哥城官方区。**2002.这是墨西哥城的保护。
21. **德国N-H，罗斯玛丽B。**2019.墨西哥一项关于临床研究中道德倾销的调查。BMC医学伦理20：38。https://doi.org/10.1186/s12910-019-0378-6.
22. **谷歌**2023.谷歌表单。
23. **格林，我，皮特，我，詹姆斯，ML。**2007.机构动物护理和使用委员会（IACUC）成员和机构官员（IO）的培训策略。ILAR J 48:131 – 142. https://doi.org/10.1093/ilar131..48.2.
24. **格林H，奥尔森IAS，Sandøe P。**2019.损害效益分析，附加值是什么？作为动物研究评估的一部分，对权衡利弊的替代策略的回顾。实验室Anim53：17-27。https://doi.org/10.1177/0023677218783004.
25. **哈雷交流。**2022.哺乳动物胚胎发生的节奏：大脑和身体发育速度的变化。大脑BehavEvol97：96-107。https://doi.org/10.1159/000523715.
26. **汉森洛杉矶。**2013.机构的动物护理和使用委员会需要更大的伦理多样性。医学伦理学39：188-190。https:// doi.org/10.1136/medethics-2012-100982.
27. **安图尼斯美联社，广州BV，桑蒂兰多尔蒂P。**2021.这是动物研究的作品。墨西哥经验。RevBioetDerecho51：99-121。https://doi.org/10.1344/ rbd2021.51.32563.
28. **侯德L，杜马斯C，Leroux T。**2003.动物伦理评价：加拿大IACUCs的观察性研究。伦理学Behav13：333-350。https://doi.org/10.1207/S15327019EB1304 2.
29. **侯德L，杜马斯C，Leroux T。**2009.伦理学：来自IACUC成员的观点。Altern实验室Anim37：291-296。https://doi .org/10.1177/026119290903700311.
30. **获得了一个。**2019.让伦理学家参与动物研究的决策制定。ILAR J 60:318 – 323. https://doi.org/10.1093/ilar/ilz023.
31. INAI。2020.Consulta Publica.在透明环境内，可以一个保护数据。
32. **约翰·霍普金斯大学彭博公共卫生学院。**动物实验的替代品中心。
33. **Khabib MNH，西瓦苏库Y，李HB，库马尔S，库CS。**2022.预测毒理学中的替代动物模型。毒理学465：153053。https://doi.org/10.1016/j.tox.2021.153053.

34．基尔肯尼，帕森斯，卡迪谢夫斯基，费MFW，卡希尔，弗莱D，赫顿J，奥特曼DG。2009．实验设计质量调查、统计分析和动物研究报告。PLoS14：e7824。 https://doi.org/10.1371/期刊。.0pone007824
35．克莱因C，巴伦AB。2016．昆虫有进行主观体验的能力。Anim感官9。 https://doi.org/ 10.51291/2377-7478.1113.
36．科普塞尔博士，鲁伊斯·德·查韦斯·MH。2015.Eticala调查。普里梅拉。生态学、编辑。墨西哥
37．路易斯·亨伯托FC。2014.这是一个大教堂的故事。拉丁美洲，22：55-76。
38．莫汉，Huneke R。2019．iacuc在负责任的动物研究中的作用。ILAR J 60:43–49. https://doi.org/10.1093/ ilar/ilz016.
39．全国研究理事会2011．实验动物的护理和使用指南。实验室Anim327：220-240。
40.NC3Rs。2022.有多少只动物被用于研究？
41.NC3Rs。2023.3卢比。
42.NC3Rs。2023.国家研究中的替代、改良和减少动物中心。
43.NORECOPA.2023.挪威的国家共识平台。
44．普卢斯，赫尔佐格H。2001．动物研究方案综述的可靠性。科学293:608–609. https://doi.org/10.1126/科学。1061621
45．罗宾逊NB、克里格K、可汗FM、霍夫曼W、张M、奈克A、永乐R、哈米德I、克里格K、吉拉迪N、瓜迪诺M。2019．目前动物模型的研究现状：一篇综述。Int J Surg 72:9–13. https://doi.org/10.1016/j.ijsu.2019.10.015.
46．Russel WMS，燃烧的RL。1960．人文实验技术的原理。MedJAust1：500-500。 https://doi.org/10.5694/j.1326-5377.1960.tb73127.x.
47．斯卡莱斯RJ，伊森伯格SB。2005.有效地使用模拟来教学和获取兽医专业人员和临床技能。J Vet Med Educ 32:461–467. https://doi.org/10.3138/jvme.32.4.461.
48．西尔弗曼J，利兹CW，克莱菲尔德J，默里A，西蒙LJ，马兰达L.2017.影响IACUC决策的因素：谁领导了讨论？《伦理道德》12：209-216。 https:// doi.org/10.1177/1556264617717827.
49．史密斯JA。1991．一个关于无脊椎动物的疼痛的问题。ILAR J 33:25–31. https://doi.org/10.1093/ilar.33.1-2.25.
50．FR，卡车司机，J，JA。1999.动物实验的伦理可接受性：一个支持决策的系统的建议。实验室Anim33：295-303。 https://doi.org/10.1258/002367799780578255.
51．州立大学。2015.Stata统计软件：第14版。大学站（TX）：StataCorp LP。
52．坦南鲍姆J，贝内特BT。2015.罗素和伯奇的3Rs当时和现在：需要清晰的定义和目的。 J 我是Assoc实验室的AnimSci54：120-132。
53.泰勒K，阿尔瓦雷斯LR。2019.对2015年全球用于科学目的的动物数量的估计。阿尔特恩实验室Anim47：196-213。 https://doi.org/10.1177/0261192919899853.
54．汤姆森A。2000．伦理学中的批判性推理：一个实用的介绍。选择牧师在线37页。 https://doi.org/10.5860/CHOICE57.37-38
55.美国农业部APHIS。2023.动物福利法案。
56．范德瓦尔克、杜赫斯特、休斯一世、阿特金森、巴尔科姆、布劳恩、加布里埃森、格鲁伯、迈尔斯、纳布、纳尔迪、范威尔根堡、津科U、祖洛J。1999.替代在高等教育中使用动物。阿尔特恩实验室Anim27：39-52。 https:// doi.org/10.1177/026119299902700105.
57．，费雷拉，C，H，摩尔EHM，范米尔PJK。2021.动物模型选择的基础是传统，而不是科学。顶点38：49-62。 https://doi.org/10.14573/顶点。2003301.
58．威廉姆斯，巴纽普。2022．人道干预点：细化终点术语，以纳入非安乐死干预选项，以改善动物福利和保持实验结果。实验室Anim56：482-489。 https://doi.org/10.1177/00236772221090801.
59．Wurbel H.2017.超过3Rs：科学有效性对动物研究的有害效益分析的重要性。实验室Anim（纽约）46：164-166。 https://doi.org/10.1038/laban..1220
60．泽马诺娃，骑士A，Lybæk S。2021．在欧洲，对动物的教育使用表明人们不愿实施替代方案。顶点38：490-506。 https://doi.org/10.14573/altex.2011111.
61．说到研究。2023.
62．雅培A。2010.巴塞尔宣言为动物研究辩护。自然468：742。 https://doi.org/10.1038/468742a.
63．佩特科夫，弗莱克内尔，墨菲K，巴索马，米切尔斯，哈蒂格R，汤普森-伊里塔尼S。2022.统一的伦理原则和动物研究的“赫尔辛基”宣言，作为国际合作的基础。神经生物库尔3：100060。 https:// doi.org/10.1016/j.crneur.2022.100060
64．史密斯，克拉顿，利利，汉森，英国大学。2018.准备：规划动物研究和试验的指南。实验室Anim 52：135141。 https://doi .org/10 . 1177/ 0023677217724823.
