# Supplementary material for: Laboratory animal ethics education improves medical students' awareness of laboratory animal ethics
Source: BMC Med Educ. 2024 Jul 1;24:709. doi: 10.1186/s12909-024-05703-9 (PMC11218205; doi:10.1186/s12909-024-05703-9)
Supplement: Supplementary file 1 — Supplementary Material 1. [file 12909_2024_5703_MOESM1_ESM.zip › The revised table/sheet001.htm]

|  |  |  |  |  |  |  |
| Learning objectives | Course contents |  | | | Course contents | Course contents |
| Cognitive objectives  To recall the laws and regulations for the protection of laboratory animals and to describe the 3R principles.  Skill objective  To correctly perform animal handling skills in grasping and fixing, correct anesthesia injection methods, culling of laboratory animals in order to reduce the pain of laboratory animals.   Attitude objectives  To treat laboratory animals in a correct animal ethical manner | Theoretical part |  | | | Cognitive objectives  To recall the laws and regulations for the protection of laboratory animals and to describe the 3R principles.  Skill objective  To correctly perform animal handling skills in grasping and fixing, correct anesthesia injection methods, culling of laboratory animals in order to reduce the pain of laboratory animals.   Attitude objectives  To treat laboratory animals in a correct animal ethical manner | Theoretical part |
| 1) the foundation, basic concepts and development process of laboratory animal ethics, with the basic principles of laboratory animal ethics, i.e., the 3R principles, as the core and entry point; 2) the laws and regulations of laboratory animal ethics and the welfare of laboratory animals; and 3) other points of knowledge such as the laboratory animal day and the euthanasia treatment of animals. |  | | | 1) the foundation, basic concepts and development process of laboratory animal ethics, with the basic principles of laboratory animal ethics, i.e., the 3R principles, as the core and entry point; 2) the laws and regulations of laboratory animal ethics and the welfare of laboratory animals; and 3) other points of knowledge such as the laboratory animal day and the euthanasia treatment of animals. |
| Practical part |  | | | Practical part |
| The core of this is the concept of correct animal ethics throughout the laboratory class. The teacher reminds the students to treat the animals gently, operate gently, eliminate animal cruelty. And strictly abide by the standardized operation procedures of animal welfare when catching and fixing the animals. When performing animal surgical operations, it is necessary to achieve the appropriate amount of anesthesia before the operation, fine operation during the operation. At the end of the experimental class, the experimental animals will be euthanasia and put into the designated location. |  | | | The core of this is the concept of correct animal ethics throughout the laboratory class. The teacher reminds the students to treat the animals gently, operate gently, eliminate animal cruelty. And strictly abide by the standardized operation procedures of animal welfare when catching and fixing the animals. When performing animal surgical operations, it is necessary to achieve the appropriate amount of anesthesia before the operation, fine operation during the operation. At the end of the experimental class, the experimental animals will be euthanasia and put into the designated location. |
|  | | |
|  | | |
|  | | | | | | |
|  | | | | | | |
|  | | | | | | |
|  | | | | | | |
|  | | | | | | |
| Learning objectives |  | | | | | |
| Cognitive objectives  To recall the laws and regulations for the protection of laboratory animals and to describe the 3R principles.  Skill objective  To correctly perform animal handling skills in grasping and fixing, correct anesthesia injection methods, culling of laboratory animals in order to reduce the pain of laboratory animals.   Attitude objectives  To treat laboratory animals in a correct animal ethical manner |  | | | | | |
|  | | | | | |
|  | | | | | |
|  | | | | | |
|  | | | | | |
|  | | | | | |
|  |  |  |
