# Supplementary material for: Laboratory animal ethics education improves medical students' awareness of laboratory animal ethics
Source: BMC Med Educ. 2024 Jul 1;24:709. doi: 10.1186/s12909-024-05703-9 (PMC11218205; doi:10.1186/s12909-024-05703-9)
Supplement: Supplementary file 1 — Supplementary Material 1. [file 12909_2024_5703_MOESM1_ESM.zip › The revised table/sheet002.htm]

| Table 2 The laboratory animal ethics awareness questionnaire |  |  |  |  |
| Questions | Options | Pre | Post | P value |
|  |  | (n=138) | (n=168) |  |
| Attitudes towards laboratory animals before and after education |  |  | | |
| 1.Whether it is necessary to oppose increased suffering of laboratory animals due | Support | 119(86.2) | 158(94.1) | <0.05 |
| to improper handling | Not supported | 19(13.8) | 10(5.9) |  |
|  |  | | |  |
| 2.Whether to support euthanasia of animals? | Support | 131(94.9) | 160(95.2) | 0.9 |
|  | Not supported | 7(5.1) | 8(4.8) |  |
| 3.Whether it is necessary to carry out activities such as silent mourning and memorials | Support | 97(70.3) | 154(91.7) | <0.05 |
| to laboratory animals? | Not supported | 41(29.7) | 14(8.3) |  |
|  |  | | |  |
| 4.Whether you support the humane treatment of laboratory animals? | Support | 133(96.4) | 166(98.8) | 0.302 |
|  | Not supported | 5(3.6) | 2(1.2 ) |  |
| Awareness of knowledge related to ethics of laboratory animals before and after education |  |  |  |  |
| 5.Whether you are aware of the relevant regulations for the protection of | Understanding | 55(39.9) | 96(57.1) | <0.05 |
| laboratory animals? | Don't know | 83(60.1) | 72(42.9) |  |
|  |  | | | |
| 6.Whether you are aware of the welfare of laboratory animals? | Understanding | 44(31.9) | 84(50.0) | <0.05 |
|  | Don't know | 94(68.1) | 84(50.0) |  |
| 7.Are you understanding of the 3R principles? | Understanding | 42(30.4) | 99(58.9) | <0.05 |
|  | Don't know | 96(69.6) | 69(41.1) |  |
| Attitudes towards in laboratory animal ethics education before and after education |  |  |  |  |
| 8.Whether you believe that strengthening animal ethics education is conducive | Support | 119(86.2) | 167(99.4) | <0.05 |
| to the development of good medical ethics? | Not supported | 19(13.8) | 1(0.6) |  |
|  |  |  | | |
| 9.Whether it is considered necessary for the school to offer courses related to ethics | Support | 102(73.9) | 133(79.2) | 0.279 |
| of laboratory animals? | Not supported | 36(26.1) | 35(20.8) |  |
|  |  |  | | |
| 10.Whether to support schools to use virtual simulation technology instead of animal | Support | 84(60.9) | 117(69.6) | 0.108 |
| experiments to reduce the number of animals used? | Not supported | 54(39.1) | 51(30.4) |  |
| \*Chi-square test; \*Significant differences when p-value<0.05. |  |  | |  |
|  |  |  |  |
