# Supplementary material for: Laboratory animal ethics education improves medical students' awareness of laboratory animal ethics
Source: BMC Med Educ. 2024 Jul 1;24:709. doi: 10.1186/s12909-024-05703-9 (PMC11218205; doi:10.1186/s12909-024-05703-9)
Supplement: Supplementary file 1 — Supplementary Material 1. [file 12909_2024_5703_MOESM1_ESM.zip › The revised table/sheet003.htm]

| Table 3 Examination paper on laboratory animal ethics | |
| Numbers | Questions |
| Q1 | World Day for Laboratory Animals is celebrated on ( )every year . |
| Q2 | If you were to euthanise a laboratory animal at the end of an animal experiment class, you would not choose ( ). |
| Q3 | If you were taking a class on animal experimentation today, in order to comply with the requirements of basic surgical techniques for animal experimentation and observe the ethics of experimental animals, you would ( ). |
| Q4 | Which of the following practices reflects the principle of "substitution" in the "3Rs" of animal ethics ( )? |
| Q5 | If you were an animal laboratory teacher and a class was to have an animal experiment lesson this morning, you would ( ). |
| Q6 | If you see a student using a mobile phone to take photos of experimental animals in an animal experiment class and post them on the Internet, what will you do ( ). |
| Q7 | Which of the following practices is not in line with the "five basic welfare" or "five freedoms" of animal ethics ( ) ? |
| Q8 | What would you do when you encounter a classmate in your neighbourhood who appears to be harming the animals purposelessly in the course of animal experiments ( ). |
| Q9 | If you are conducting a laboratory class on appendectomy in rabbits, and you find that the rabbits show signs of resuscitation after anaesthesia, you would ( ). |
| Q10 | If you are a small assistant in the animal laboratory of the university, in order to ensure the welfare of experimental animals, you will ( ). |
|  |  |
